# Compact dual-slot antenna with enhanced bandwidth for multi-band wireless and satellite systems

**DOI:** 10.1038/s41598-026-45265-y

**Published:** 2026-04-01

**Authors:** B. R. Shivakumar, M. Pallavi

**Affiliations:** 1 Nitte (Deemed to Be University), Department of ECE, NMAM Institute of Technology, Nitte, Udupi India; 2 Manipal Institute of Technology, Manipal Academy of Higher Education, Manipal, Karnataka 576104 India

**Keywords:** Compact antenna design, Dual-slot antenna, GPS and ADS-B applications, Ku-band satellite communication, MIMO antenna, Mutual coupling reduction, Multi-band resonance, Orthogonally placed radiators, X-band radar, Engineering, Physics

## Abstract

This paper presents a compact, orthogonally placed 2-port MIMO antenna optimized for next-generation wireless and satellite communication systems. A key challenge in MIMO antenna design—mutual coupling between radiating elements—is addressed through an effective decoupling strategy, achieving isolation levels better than − 30 dB across the entire operating band. The proposed antenna exhibits dual resonances: the first at 1.6 GHz, covering a wide frequency range from 0.7 GHz to 8.6 GHz with a fractional bandwidth of 170.1%, and the second at 12.6 GHz, covering 12.0 GHz to 15.4 GHz with a fractional bandwidth of 24.82%. These bands collectively support applications such as GPS, ADS-B, X-band radar, and Ku-band satellite communications. The antenna demonstrates a peak gain of 4.9 dB with an overall footprint of only $0.2 \times 0.2 \times 0.002\lambda^{3}$ at 1.6 GHz. A detailed performance evaluation—including isolation, far-field radiation patterns, realized gain, envelope correlation coefficient (ECC), mean effective gain (MEG), and total active reflection coefficient (TARC)—confirms the antenna’s wideband operation, low correlation, and suitability for high-capacity, interference-resilient MIMO systems.

## Introduction

Ultra-Wideband (UWB) antennas are a key enabler of modern wireless systems, offering an exceptionally wide operational bandwidth while maintaining a low power spectral density to minimize interference with coexisting communication services. This wide bandwidth supports high-speed data transmission, centimetre-level positioning accuracy (typically within ± 1 cm to ± 10 cm), and high-resolution imaging capabilities^1–3^. The broad spectral coverage of UWB antennas makes them suitable for a diverse range of applications, including 5G, automotive radar, IoT, industrial automation, indoor navigation, and medical imaging, where a single radiator can simultaneously support sensing, communication, and tracking functionalities. Their combination of high precision, large capacity, robustness in multipath environments, and hardware efficiency positions them as a critical component for current and emerging wireless systems. The adoption of UWB technology accelerated following the FCC authorization for practical, unlicensed operation in the 3.1–10.6 GHz frequency band, although with a power limitation of less than –41.3 dBm/MHz.

The constraint on transmit power can be mitigated by increasing channel capacity, which is achievable through MIMO technology^4^. By leveraging spatial multiplexing, MIMO systems significantly enhance overall channel performance, including improved SNR and increased data throughput^5–8^. However, the integration of UWB systems with MIMO architectures introduces additional design challenges, as performance is strongly influenced by factors such as impedance bandwidth, inter-element isolation, profile height, gain, miniaturization, and radiation stability^9^. Among these, compactness is a primary consideration in the design of integrated communication systems. In UWB MIMO antennas, compactness can be achieved by reducing radiator dimensions, decreasing inter-element spacing, or arranging radiators orthogonally. However, reduced spacing often results in higher mutual coupling, leading to EMI and degradation of radiation characteristics.

To address these challenges, various isolation enhancement techniques have been explored in the literature, aiming to improve bandwidth, isolation, and overall antenna performance. A compact 2-port U-shaped patch antenna ($0.8 \times 0.4 \times 0.002\lambda^{3}$) for UWB applications was presented in^10^, where a ML EBG structure was placed vertically between the radiators to mitigate E-plane coupling. In^11^, an irregular polygon-shaped 2-port antenna ($0.2 \times 0.4 \times 0.019\lambda^{3}$at 3.5 GHz) designed for 5G NR employed a T-shaped decoupling structure between horizontally spaced radiators to improve both isolation and bandwidth. For 5G mmWave applications^12^, proposed a 2-port horizontally structured MIMO antenna incorporating an MTM decoupler, achieving dual resonances at 24 GHz and 41 GHz with FBW values of 41.02% and 18.59%, respectively. Similarly^13^, introduced an asymmetrical U-shaped 2-port MIMO antenna for UWB applications, where adequate radiator spacing combined with optimized ground-plane modification yielded a wide bandwidth (3.10–11.85 GHz) and high isolation without additional decoupling elements.

At mmWave frequencies^14^, reported a 2-port MTM-loaded MIMO antenna ($0.9 \times 1.7 \times 0.07\lambda^{3}$ at 28 GHz) featuring dual resonances at 28 GHz (FBW = 18.18%) and 38 GHz (FBW = 10.85%). An orthogonally arranged 2-port MIMO antenna ($0.9 \times 0.9 \times 0.14\lambda^{3}$) for mmWave operation was presented in^15^, utilizing a stair-shaped decoupling structure to achieve a single resonance at 28 GHz with an FBW of 16.69% and an isolation improvement up to –32 dB across the operating spectrum.

In these works, multiple isolation enhancement strategies have been demonstrated, including the introduction of NLs between UWB MIMO feeds^16–24^, the placement of parasitic patches of varying geometries in proximity to radiators^25–29^, the etching of optimally shaped slits and slots between radiators and on the ground plane^30–33^, the use of defected and partial ground structures^34–36^, and the integration of artificial electromagnetic structures between radiators and within the ground plane^12,37,38^. These methods collectively address the mutual coupling challenge, enabling compact UWB MIMO designs with enhanced performance for next-generation wireless systems.

This study proposes a compact two-port MIMO antenna with integrated band-notch features, specifically tailored for UWB operations. The radiative elements are arranged orthogonally and separated by a 45°-tilted T-shaped parasitic strip, thereby enhancing polarization diversity and inter-element isolation. Unlike traditional centre-fed radiator structures, offset-fed arrangements are utilized for both radiators to produce a much broader impedance bandwidth. In addition, asymmetrical slots are etched on the ground plane to extend the operational bandwidth while providing the appropriate band-rejection capability. The MIMO diversity functions for the proposed structure remains consistent with the theoretical bounds and the optimized design has a modest footprint of $0.2 \times 0.2 \times 0.002{\uplambda }^{3} \left( {38.5 \times 38.5 \times 0.5{\mathrm{mm}}^{3} } \right)$ making it ideal for integration with modern wireless systems. The subsequent components constitute an in-depth discussion of the antenna design approach, detailing the structural layout and optimization methods used. In addition, both simulated and empirical results are shown to verify the proposed layout, emphasizing its performance features and verifying the precision of numerical evaluation using measurement-based validation.

## Design configuration and analysis of the proposed mimo antenna

This section discusses the various evolution stages of the proposed 2-port MIMO antenna, the significance of the different components of the proposed design, and the parametric analysis carried out to fix the dimension of the most significant parts in the proposed design.

### Single-element antenna design

**Fig. 1:**
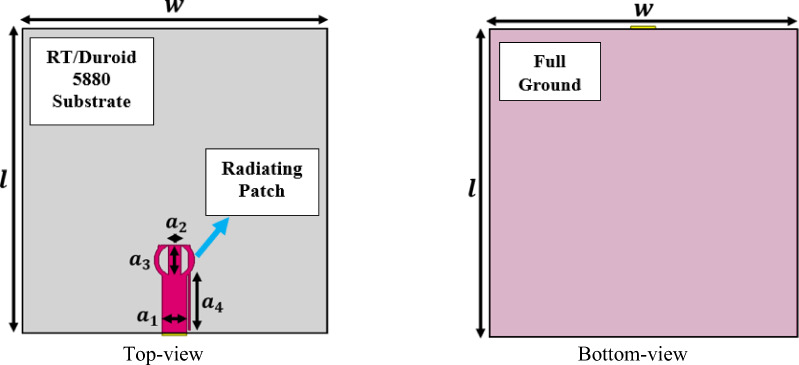
Proposed single-element with full ground plane.

The proposed single-element antenna structure is illustrated in Fig. 1. The antenna is constructed on a 0.5 mm thick RT/Duroid 5880 substrate that has a relative permittivity of 2.2 and a dielectric loss tangent of 0.0009. The structure measures $38.5 \times 38.5{\text{ mm}}^{2}$ ($0.2 \times 0.2 \times 0.002{\uplambda }^{3}$ at 1.6 GHz), making it perfect for wireless platforms with limited space.

**Fig. 2:**
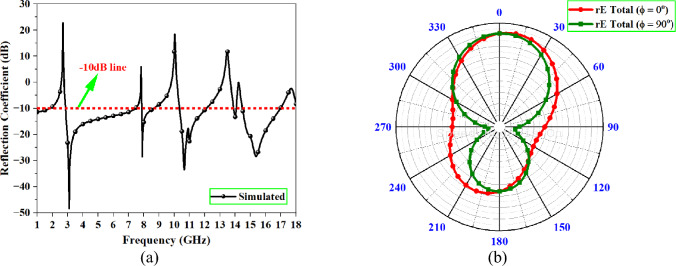
Simulated recording for the single-element antenna (**a**) ${\mathrm{S}}_{11}$ plot (**b**) Radiation pattern.

Figure 2 depicts the simulated attributes of the proposed single-element antenna, such as its ${\mathrm{S}}_{11}$ response and far-field radiation performance. In Fig. 2a, the ${\mathrm{S}}_{11}$ parameter is analysed across the 1—18 GHz spectrum, with numerous significant resonant modes near 2.4 GHz, 3.2 GHz, 7 GHz, 10 GHz, and 15 GHz. These resonances reflect the inherent multiband behaviour of the radiator, resulting in efficient impedance-matched bandwidths of around 2.5—4.5 GHz, 6.5—7.5 GHz, 10—11 GHz, and 14—15 GHz. Figure 2b shows radiation patterns for $\phi = 0^\circ$ and $\phi = 90^\circ$, indicating stable and well-defined far-field radiation properties. Both principal-plane patterns show a bidirectional, figure-eight-shaped distribution, indicating significant radiation at $0^\circ$ and $180^\circ$ but lower radiation at orthogonal angles.

### Resonant frequency model for the fundamental patch

The resonant frequency of a rectangular patch antenna using the dominant ${\mathrm{TM}}_{10}$ mode is:

1 $$f_{r} = \frac{c}{{2L_{eff} \sqrt {\varepsilon_{eff} } }}$$

where, ${\mathrm{c}}$ is the speed of light ($3 \times 10^{8} {\mathrm{m}}/{\mathrm{s}}$), ${\mathrm{L}}_{{{\mathrm{eff}}}}$ represents the effective length, and ${\upvarepsilon }_{{{\mathrm{eff}}}}$ represents the effective permittivity. In patch antenna structures, the fringing field generates an effective dielectric constant, which is mathematically expressed by

2 $${\upvarepsilon }_{{{\mathrm{eff}}}} = \frac{{{\upvarepsilon }_{{\mathrm{r}}} + 1}}{2} + \frac{{{\upvarepsilon }_{{\mathrm{r}}} - 1}}{2}\left( {1 + 12\frac{{\mathrm{h}}}{{{\mathrm{W}}_{{{\mathrm{eq}}}} }}} \right)^{ - 1/2}$$

where, ${\mathrm{W}}_{{{\mathrm{eq}}}}$ denotes the equivalent patch width which approximates the scattered current region.

For irregular geometries (like the proposed structure), ${\mathrm{W}}_{{{\mathrm{eq}}}}$ is taken as

3 $${\mathrm{W}}_{{{\mathrm{eq}}}} = \frac{{{\mathrm{A}}_{{{\mathrm{patch}}}} }}{{{\mathrm{L}}_{{{\mathrm{max}}}} }}{ }$$

where ${\mathrm{A}}_{{{\mathrm{patch}}}}$ represents the total conductor area and ${\mathrm{L}}_{{{\mathrm{max}}}}$ represents the maximum current path length from the feed point to the top of the patch. Any planar radiator, regardless of shape, will resonate if its electrical length matches the following conditions:

4 $${\mathrm{L}}_{{{\mathrm{eff}}}} \approx \frac{{{\uplambda }_{{\mathrm{g}}} }}{2}{ }$$

where, ${\uplambda }_{{\mathrm{g}}}$ is the guided wavelength and it is given by:

5 $${\uplambda }_{{\mathrm{g}}} = { }\frac{{\mathrm{c}}}{{{\mathrm{f}}\sqrt {{\upvarepsilon }_{{{\mathrm{eff}}}} } }}{ }$$

There, the expected resonance frequency of the dominant mode is:

6 $${\mathrm{f}}_{{\mathrm{r}}} = \frac{{\mathrm{c}}}{{{\mathrm{fL}}_{{{\mathrm{eff}}}} \sqrt {{\upvarepsilon }_{{{\mathrm{eff}}}} } }}{ }$$

In the proposed structure, ${\mathrm{L}}_{{{\mathrm{eff}}}}$ represents the overall length of the folded meander-slot structure (antenna structure), which explains why multiple resonances emerge in the 1 to 16GHz frequency range as illustrated in Fig. 2a.

The proposed antenna structure consists of hollow sections or vertical slots, identical side stubs, and a central feed protrusion. Each discontinuity causes a localized current change, that can be represented as a transmission-line stub with impedance:

7 $${\mathrm{Z}}_{{{\mathrm{stub}}}} = - {\mathrm{jZ}}_{0} \cot \left( {{\upbeta }\ell } \right){ }$$

where, $\ell$ denotes the slot length/physical stub, ${\upbeta }$ is the coupling factor ($\frac{{2{\uppi }}}{{{\uplambda }_{{\mathrm{g}}} }}$), resonance occurs when, $\beta l = n\pi$ (where ${\mathrm{n}} = 1,{ }2,{ }3,..$). This justifies the multi-band behaviour and several ${\mathrm{S}}_{11}$ dips observed in the plot.

The impedance bandwidth of a slot-influenced wideband radiator is given by:

8 $${\mathrm{BW}} \approx \frac{1}{{\mathrm{Q}}} = \frac{{{\mathrm{R}}_{{\mathrm{r}}} }}{{{\upomega }\left( {{\mathrm{L}}_{{{\mathrm{eq}}}} - \frac{{{\mathrm{C}}_{{{\mathrm{eq}}}}^{ - 1} }}{{{\upomega }^{2} }}} \right)}}{ }$$

Multiple resonant routes produce numerous low-Q resonances, resulting in the observed wideband behaviour.

The patch width (${\mathrm{W}}$), fringing field length extension ($\Delta L$), and effective length (${\mathrm{L}}$) is given by:

9 $${\mathrm{W}} = \frac{{\mathrm{c}}}{{2{\mathrm{f}}_{{\mathrm{r}}} }}\sqrt {\frac{2}{{{\upvarepsilon }_{{\mathrm{r}}} + 1}}} { }$$

10 $$\Delta L = 0.412h\frac{{\left( {\varepsilon_{eff} + 0.3} \right)\left( {\frac{W}{h} + 0.264} \right)}}{{\left( {\varepsilon_{eff} - 0.258} \right)\left( {\frac{W}{h} + 0.8} \right)}}$$

11 $$L_{eff} = L + 2\Delta L$$

where, ${\mathrm{L}}$ is the physical length of the patch.

**Fig. 3:**
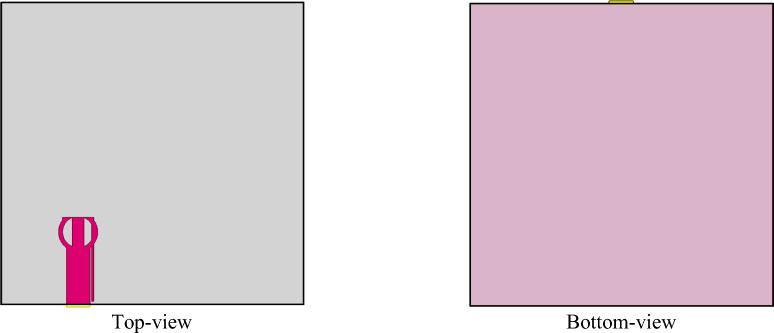
Proposed Offset-Placed Single Element Antenna with Full Ground Plane.

To construct a highly miniaturized two-port antenna arrangement, the single radiating element is relocated laterally towards the left edge of the substrate as illustrated in Fig. 3.

**Fig. 4:**
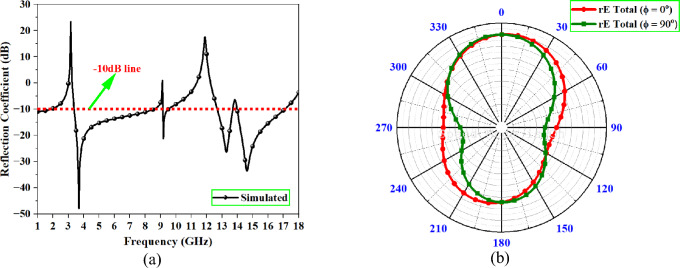
Simulated recording for the offset-placed single-element antenna (**a**) ${\mathrm{S}}_{11}$ plot (**b**) Radiation pattern.

Relocating the patch toward the left side of the substrate triggers the two characteristic changes: (1) the shift in the ${\mathrm{S}}_{11}$ plot and the resonance depths/bandwidths change, and (2) the far-field co-polar pattern becomes asymmetric and slightly slanted, and these two changes are visible in Fig. 4a and b. Both changes stem from the same physical cause: the radiator no longer sees the same electrical border conditions and coupling setting as when centered.

When the radiating patch is shifted by a distance ‘d’ from the centre of the substrate, its proximity to the conducting boundary changes, modifying the effective electrical length of the antenna. This perturbation may be approximated as follows:

12 $$L_{eff, offset} \approx L_{eff } \left( {1 + \kappa e^{{ - \frac{\gamma d}{h}}} } \right)$$

where, ‘${\upkappa }$’ and ‘${\upgamma }$’ both are empirical parameters based on antenna structure and substrate dielectric.

Furthermore, higher-order resonances show significant shifts in both frequency locations and return-loss magnitudes. Higher-order modes exhibit this behaviour because they are naturally more sensitive to boundary discontinuities, radiating element lateral movement, and ground plane asymmetry, which are clearly illustrated in Fig. 4a.

### Evolution of two – port antenna structure

**Fig. 5:**
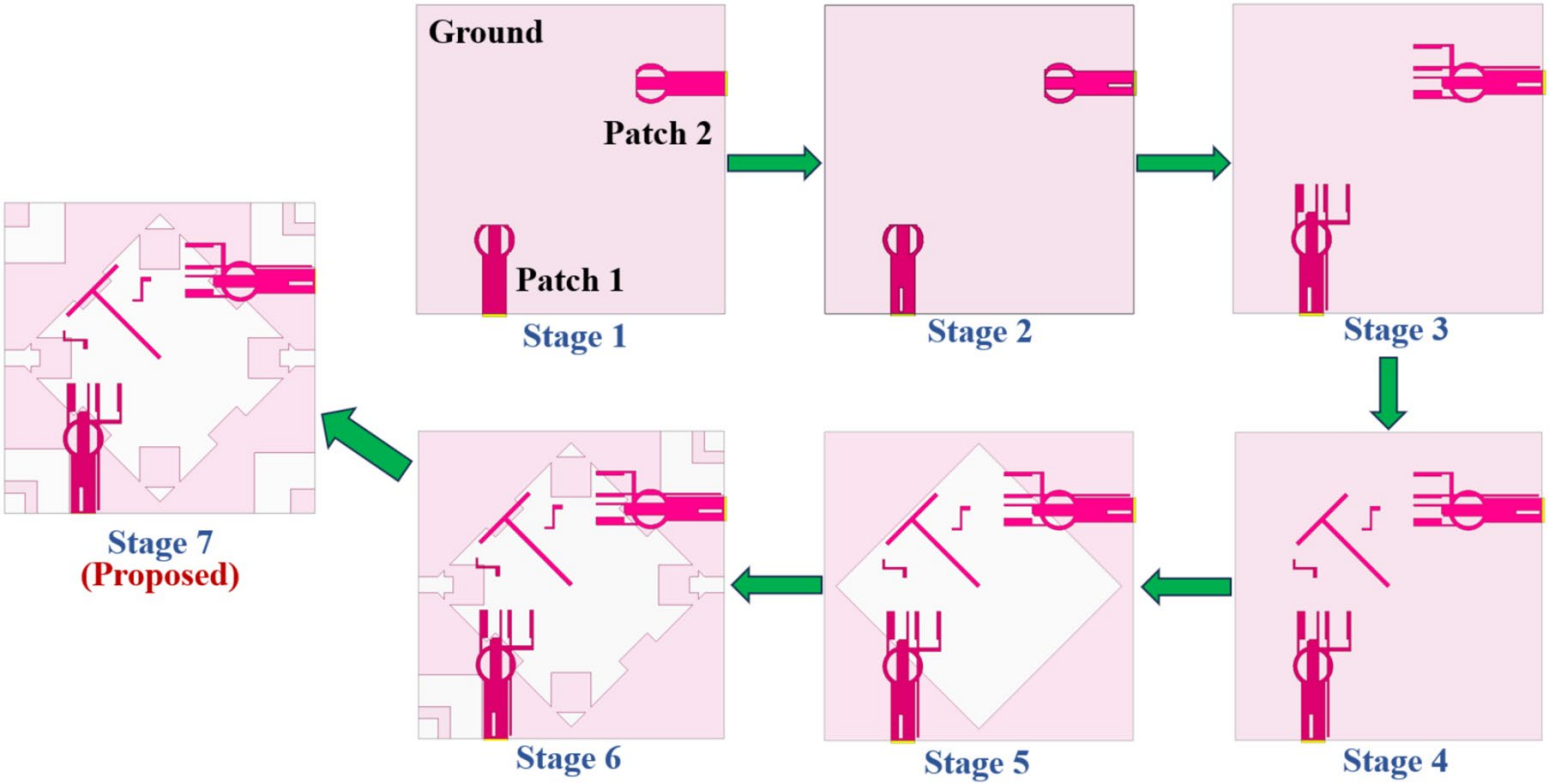
Design progression of the proposed 2-port MIMO antenna.

The antenna geometry was iteratively tailored throughout many design phases to accomplish UWB performance with an optimum reflection coefficient and better antenna characteristics. Figure 5 depicts the sequential flow of the proposed antenna through seven critical development phases.

**Fig. 6:**
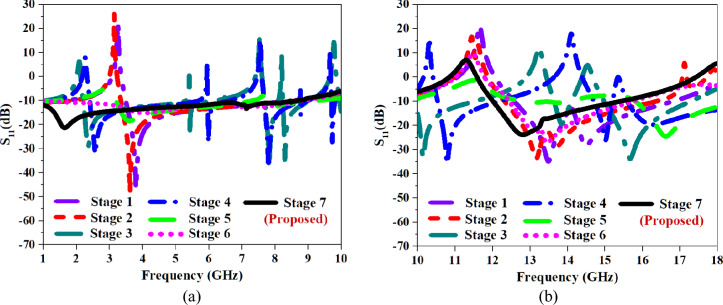
Reflection Coefficient (${\mathrm{S}}_{11}$) plot at various design progression stages (**a**) ${\mathrm{S}}_{11}$ response from 1–10GHz (**b**) ${\mathrm{S}}_{11}$ response from 10–18GHz.

In Stage 1, two simple symmetrical radiating patches are placed orthogonally on a fully copper-coated ground plane. This initial technique only enabled narrowband resonance at a single frequency. As illustrated in Fig. 6, the RC for the proposed geometry is above −10dB for the majority of the frequency spectrum (Fig. 6a and b), indicating that the geometry has poor impedance matching and, as a result, a limited operational bandwidth. The feeding structure was altered in Stage 2 by etching rectangular slots. The slot insertion effectively increased the length of the current path, contributing to a marginal increase in impedance bandwidth and facilitating multiple resonances by improving impedance matching. Nonetheless, the induced modes remained poorly coupled and substantially isolated, resulting in inadequate modal interaction. This inhibits the ability of the radiator to achieve efficient and seamless wideband spectrum.

In Stage 3, additional slots and parasitic elements are etched in close proximity to the radiators, which interrupt the surface current distribution and facilitates a number of resonant modes, enabling multiband operation. Although this structure effectively produces new resonances, impedance matching across the working spectrum is skewed, limiting bandwidth homogeneity and diminishing wideband performance. To improve coupling between adjacent resonant modes and redistribute the surface current, more diagonal or angular parasitic elements are positioned near the radiators in Stage 4. Although the impedance bandwidth and multi-resonance behaviour are improved by this structural modification, there are still apparent impedance mismatches at certain frequencies and an abrupt transition between resonances.

As shown in Stage 5, the ground plane is etched with Rotated Square Defected Ground Structure (RS-DGS) to enhance structural symmetry and current flow, hence stabilising the impedance characteristics. Additionally, the altered ground plane helps to increase radiation efficiency. In comparison to the prior design stages, the antenna thus obtains a better broad response with more consistent impedance matching. Additional slits, slots, and parasitic elements are placed in the ground surface to enhance the antenna performance in Stage 6. The incorporation of these has introduced capacitive-inductive coupling by minimising surface waves, resulting in improved bandwidth and isolation.

The Stage 7 is the final optimised structure, in which multiple enhancement techniques are combined—the rotated substrate layout improves symmetry and current distribution, the ground shaping suppresses surface waves while stabilising impedance, and slots and parasitic elements introduce multiple resonances. The final layout has improved the isolation and performance, resulting in a smoother and wider UWB response (${\mathrm{S}}_{11} < - 10{\mathrm{dB}}$ across the band), making it more appropriate for sophisticated UWB/MIMO/5G applications.

### Antenna structure and analysis

**Fig. 7:**
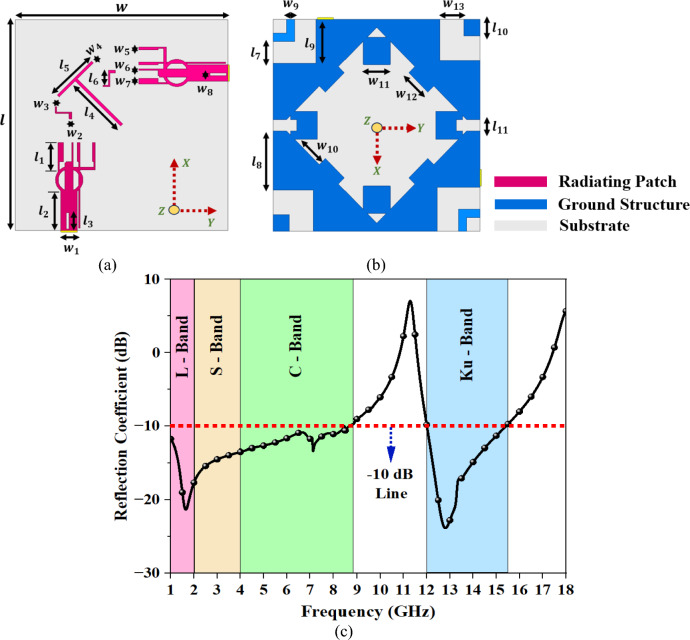
Proposed two-port antenna structure with simulated result (**a**) Radiating patch (Top-view).(**b**) Defected ground structure (**c**) Simulated ${\mathrm{S}}_{11}$ (RC) plot for the proposed antenna.

Figure 7 displays the proposed antenna layout, with Fig. 7a representing the dual radiating elements and Fig. 7b representing the ground plane and Fig. 7c showing the Simulated ${\mathrm{S}}_{11}$ plot for the proposed antenna demonstrating wideband impedance matching with notched bands, validated against the $- 10{\mathrm{dB}}$ reference line across L-, S-, C-, and Ku-bands.

**Fig. 8:**
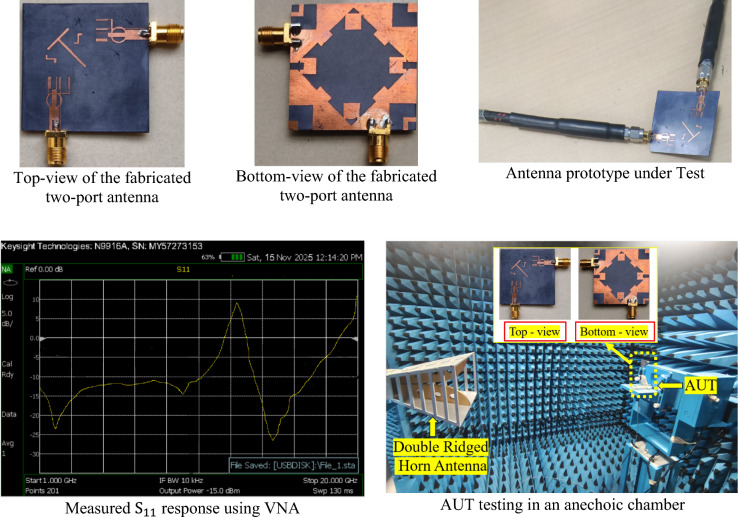
Prototype of the Developed Antenna and the Measurement Setup.

The proposed structure comprising two identical radiating patches that are placed orthogonally to generate polarization diversity while minimizing mutual interaction. A parasitic T-shaped strip is strategically located between the two radiators, acting as a decoupling structure to further reduce coupling currents and improve isolation performance. The radiators are powered by microstrip lines, and the polygonal patch topology offers wideband impedance compliance. The ground plane is carefully engineered with partial slots and shaping, which aid in suppressing surface waves and improving impedance matching across the desired band. Through the combined effect of radiator orientation, parasitic decoupling, and ground modification, the antenna achieves wideband impedance bandwidth with ${\mathrm{S}}_{11} < - 10{\mathrm{dB}}$, consistent isolation, and optimum current distribution by combining radiator orientation, parasitic decoupling, and ground modification, Fig. 8.

The integrated design offers a consistent UWB responses, compactness, and improved isolation, rendering the antenna ideal for MIMO, UWB, and 5G communication systems. The proposed antenna is developed on 0.5mm thick RT/Duroid 5880 and its fabricated prototype (top and bottom views), its analysis using a VNA for ${\mathrm{S}}_{11}$ characterisation, and the far-field assessment of the antenna under test (AUT) inside an anechoic chamber with a double-ridged horn antenna. The designed parameter of the proposed structure is summarized in Table 1.

**Table 1:** Design parameters for proposed structures (DP: Design Parameters, D: Dimensions).

DP	D (mm)	DP	D (mm)	DP	D (mm)	DP	D (mm)	DP	D (mm)
$a_{1}$	3	${\mathrm{w}}_{2}$	0.5	${\mathrm{w}}_{9}$	1.5	${\mathrm{l}}_{3}$	3	${\mathrm{l}}_{10}$	3
$a_{2}$	1.5	${\mathrm{w}}_{3}$	0.25	${\mathrm{w}}_{10}$	6.5	${\mathrm{l}}_{4}$	11.6	${\mathrm{l}}_{11}$	2
$a_{3}$	3.7	${\mathrm{w}}_{4}$	0.5	${\mathrm{w}}_{11}$	5	${\mathrm{l}}_{5}$	8.8		
$a_{4}$	7.25	${\mathrm{w}}_{5}$	0.5	${\mathrm{w}}_{12}$	5	${\mathrm{l}}_{6}$	2.75		
${\mathrm{w}}$	38.5	${\mathrm{w}}_{6}$	0.25	${\mathrm{w}}_{13}$	4.5	${\mathrm{l}}_{7}$	4		
${\mathrm{l}}$	38.5	${\mathrm{w}}_{7}$	1	${\mathrm{l}}_{1}$	5.35	${\mathrm{l}}_{8}$	10.75		
${\mathrm{w}}_{1}$	3	${\mathrm{w}}_{8}$	0.5	${\mathrm{l}}_{2}$	7.25	${\mathrm{l}}_{9}$	8		

### Modal analysis

The Modal analysis (MA) is critical in antenna design because it offers a physics-based comprehension regarding how an antenna transmits by breaking down surface currents into a collection of Characteristic Modes (CM). Modal analysis provides insight into the radiator behaviour, unlike standard parametric tuning, which just determines whether S₁₁ fits criteria. Each characteristic phase has a unique resonance frequency, radiation pattern, current distribution, and contribution to overall radiated power. By examining these modes, designers can determine which modes are effective for radiation, that are non-radiating or negate each other out, and how geometrical changes activate or repress specific modes. This approach is particularly useful in wideband, multiband, and MIMO antennas, where many resonances and current pathways interact in complex ways.

The MA is conceptually based on solving the following eigenvalue expression extracted from the MoM impedance matrix:

13 $${\mathrm{XJ}}_{{\mathrm{n}}} = {\uplambda }_{{\mathrm{n}}} {\mathrm{RJ}}_{{\mathrm{n}}}$$

where, ${\mathrm{X}}$ and ${\mathrm{R}}$ represent the imaginary and real parts of impedance operator (reactive energy and radiated power), ${\mathrm{J}}_{{\mathrm{n}}}$ is the ${\mathrm{n}}^{{{\mathrm{th}}}}$ mode eigen current, ${\uplambda }_{{\mathrm{n}}}$ is the eigenvalue characterizing the resonance condition.

When ${\uplambda }_{{\mathrm{n}}} = 0$, a mode is in resonance. A mode contributes strongly when ${\mathrm{MS}}_{{\mathrm{n}}} \approx 1$, a mode makes a significant contribution due to its low reactive energy and effective radiation. To estimate the electromagnetic impact of each structural element in the proposed two-port antenna, a comprehensive MA was performed. The characteristic angle (CA), eigenvalue (EV), and modal significance (MS) were assessed at the two primary working frequencies—1.6 GHz and 12.6 GHz, for all seven evolutionary stages of the system, as shown in Fig. 5.

**Fig. 9:**
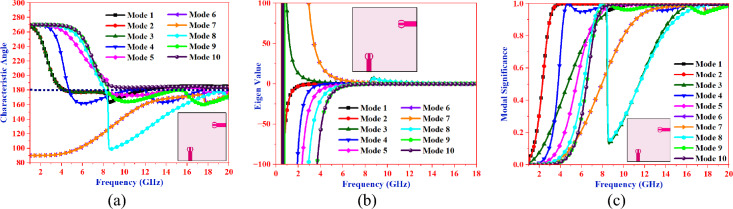
Modal analysis results for Stage 1 at 1.6 GHz (**a**) Characteristic Angle (**b**) Eigenvalue. (**c**) Modal Significance.

The MA for all Stages is performed at two representative frequencies, 1.6 GHz and 12.6 GHz, to understand how different characteristic modes contribute to radiation across the wide operating bands. In Stage 1 (Fig. 9), at 1.6GHz, the CA plot (Fig. 9a) implies that only a handful of lower-order modes reach the resonant condition (i.e., CA at 180°), confirming that the radiation is driven predominantly by fundamental currents flowing through radiator 1. The EV (Fig. 9b) and MS plots (Fig. 9c) show that certain modes traverse the zero-eigenvalue zone and have values close to unity. These findings demonstrate that Stage 1 promotes substantial low-frequency resonance via a limited set of dominant modes.

**Fig. 10:**
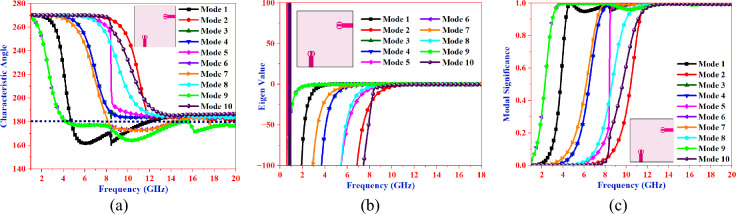
Modal analysis curve for Stage 1 at 12.6GHz (**a**) Characteristic Angle (**b**) Eigen Value (**c**) Modal Significance.

In contrast, at 12.6 GHz (Fig. 10), the modal pattern shifts substantially. The CA (Fig. 10a) demonstrate that many higher-order modes have now reached resonance, indicating that the antenna is transitioning to more complicated current distributions ideal for higher-band performance. The EVs (Fig. 10b) of these modes approach zero in this frequency range, while the accompanying MS curves (Fig. 10c) rise strongly toward unity, suggesting efficient radiation from many active modes. This change suggests that the Stage 1 geometry is intrinsically multiband, with various modes governing radiation at lower and higher frequencies. The integrated MA provides a clear physical understanding of how the antenna transitions from fundamental to higher-order resonant modes as frequency increases.

**Fig. 11:**
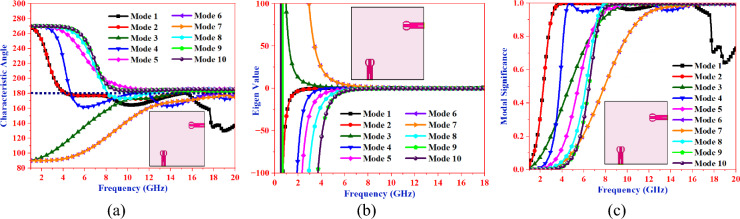
Modal analysis results for Stage 2 at 1.6 GHz (**a**) Characteristic Angle (**b**) Eigenvalue. (**c**) Modal Significance.

As illustrated in Fig. 11, at 1.6 GHz, the Stage 2 geometry changes the dominant current routes owing to the asymmetric slot layout, resulting in just a few lower-order modes approaching the 180° CA resonance (Fig. 11a). The EV plot (Fig. 11b) reveals that just the primary mode crosses zero around this frequency, indicating significant excitation of a single resonant mode. The MS (Fig. 11) shows a single peak at unity, while all other modes stay below the 0.707 threshold. Thus, at 1.6 GHz, Stage 2 functions mostly as a single-mode radiator, with improved modal isolation and decreased higher-order contributions.

**Fig. 12:**
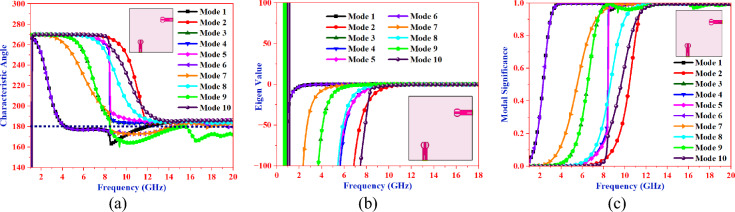
Modal analysis results for Stage 2 at 12.6 GHz (**a**) Characteristic Angle (**b**) Eigenvalue. (**c**) Modal Significance.

At 12.6 GHz (Fig. 12), the Stage-2 alteration activates multiple higher-order modes, as evidenced by various CA curves (Fig. 12a) that converge on the 180° resonance region. The related EV trajectories (Fig. 12b) exhibit multiple zero-crossings, indicating substantial multimode resonance. The MS values (Fig. 12c) greater than 0.707 for many modes confirm that Stage 2 acts as a multimode radiator at this frequency, allowing for a wider bandwidth and deeper field interactions.

**Fig. 13:**
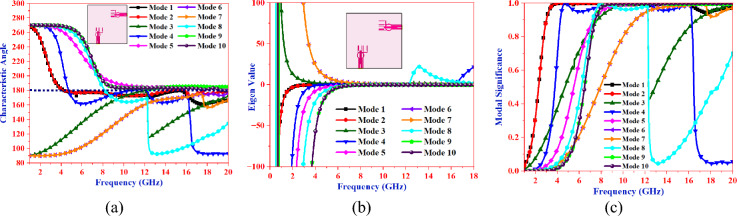
Modal analysis results for Stage 3 at 1.6 GHz (**a**) Characteristic Angle (**b**) Eigenvalue. (**c**) Modal Significance.

In Stage 3 (Fig. 13), further changes in the radiator alter the surface-current pattern, allowing just a few lower-order modes to meet the 180° CA (Fig. 13a), criterion near 1.6 GHz. The EV plot (Fig. 13b), reveals that only one dominant mode crosses zero, suggesting strong single-mode excitation, while all other modes maintain large EVs with poor coupling. Similarly, the MS (Fig. 13c), shows a single spike close to unity, indicating that the antenna emits primarily via single stable and clearly defined characteristic mode at this frequency.

**Fig. 14:**
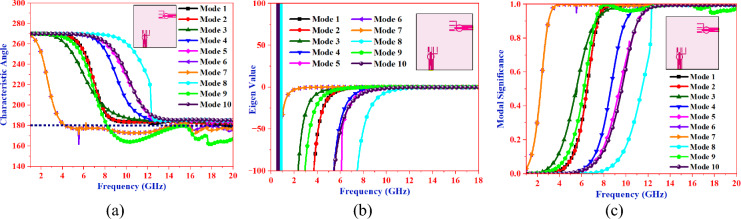
Modal analysis results for Stage 3 at 12.6 GHz (**a**) Characteristic Angle (**b**) Eigenvalue. (**c**) Modal Significance.

As shown in Fig. 14, at 12.6 GHz, structural modifications allow for a greater onset of several higher-order modes, with various CA curves (Fig. 14a) approaching the 180° resonance region. The EV characteristics (Fig. 14b) also show several zero-crossings, implying that several modes become effectively excitable and contribute to radiation. This is mirrored in the MS (Fig. 14c), which shows that many modes exceed the 0.707 threshold, indicating a multimode radiator behaviour.

**Fig. 15:**
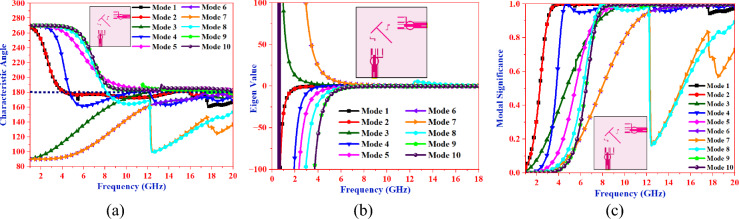
Modal analysis results for Stage 4 at 1.6 GHz (**a**) Characteristic Angle (**b**) Eigenvalue. (**c**) Modal Significance.

In Stage 4 (Fig. 15), the insertion of T-shape parasitic element significantly affects the surface-current structure, leaving only few lower-order modes nearing the 180° CA resonance (Fig. 15a) at 1.6 GHz. The EV characteristics (Fig. 15b) show that only the fundamental mode has a zero-crossing in this region, supporting strong single-mode emission. The MS profile (Fig. 15c) likewise displays a prominent MS peak near unity, with all other modes remaining below the excitation threshold, indicating that Stage 4 is primarily a single-mode radiator at the lower resonance.

**Fig. 16:**
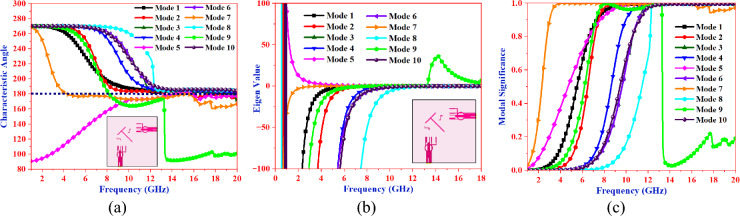
Modal analysis results for Stage 4 at 12.6 GHz (**a**) Characteristic Angle (**b**) Eigenvalue. (**c**) Modal Significance.

At 12.6 GHz (Fig. 16), the addition of T structure facilitates the activation of numerous higher-order modes, with many CA curves (Fig. 16a) reaching the 180° resonant state. This is corroborated by several EV zero-crossings (Fig. 16b), which indicate significant modal coupling and increased excitation of higher-order currents. The MS curves (Fig. 16c) show that numerous modes exceed the 0.707 threshold, indicating a definite multimode radiation mechanism and a larger electromagnetic consequence at higher frequencies.

**Fig. 17:**
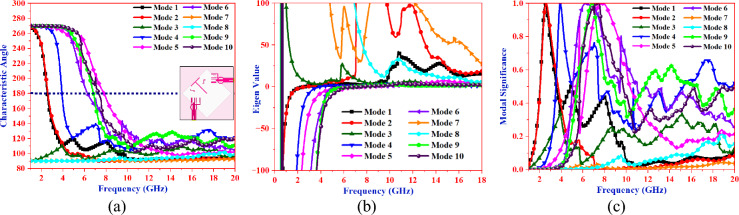
Modal analysis results for Stage 5 at 1.6 GHz (**a**) Characteristic Angle (**b**) Eigenvalue. (**c**) Modal Significance.

In Stage 5 (Fig. 17), the 45° tilted square etching on the ground plane has significantly redistributes the return-current flow, allowing only a few lower-order modes to approach the 180° CA resonance (Fig. 17a) at 1.6 GHz. The EV plot (Fig. 17b) shows that only one dominant mode crosses zero, suggesting significant but isolated modal excitation, while all other modes remain weakly coupled. Similarly, the MS response (Fig. 17c) has only one noticeable peak near unity, indicating that the radiator behaves primarily as a single-mode emitter at the lower frequency despite the complex disruption.

**Fig. 18:**
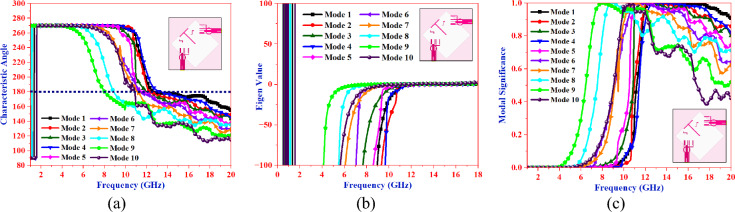
Modal analysis results for Stage 5 at 12.6 GHz (**a**) Characteristic Angle (**b**) Eigenvalue. (**c**) Modal Significance.

At 12.6 GHz (Fig. 18), etching the slot improves the stimulation of many higher-order modes by changing current restriction and enabling new resonance routes. The CA curves (Fig. 18a) reveal various modes converging into the 180° region, while the EV response (Fig. 18b) shows multiple zero-crossings, indicating strong multimode resonance. The MS plots (Fig. 18c) show numerous modes with MS > 0.707, showing that Stage 5 acts as a discrete multimode radiator at higher frequencies.

**Fig. 19:**
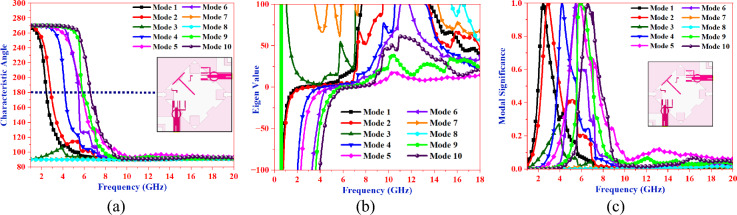
Modal analysis results for Stage 6 at 1.6 GHz (**a**) Characteristic Angle (**b**) Eigenvalue. (**c**) Modal Significance.

In Stage 6 (Fig. 19), at 1.6 GHz, the introduction of the defective ground structure (DGS) changes current flow pattern, leading some CA (Fig. 19a) towards the resonance condition (180°) more gradually than in previous stages. The EV distribution (Fig. 19b) demonstrates a better divergence among dominant and non-dominant modes, implying greater modal controllability. Similarly, the MS curves (Fig. 19c) show sharper peaks around the working region, indicating higher excitation of important radiating modes due to changed ground currents.

**Fig. 20:**
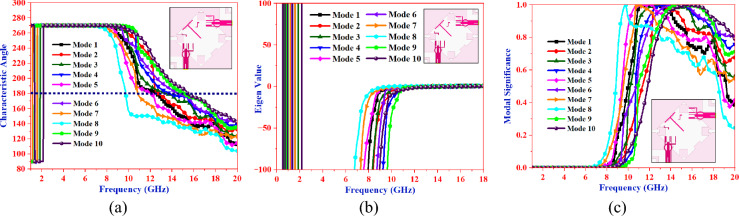
Modal analysis results for Stage 6 at 12.6 GHz (**a**) Characteristic Angle (**b**) Eigenvalue. (**c**) Modal Significance.

At 12.6 GHz (Fig. 20), the DGS allows for smoother modal transitions by suppressing undesirable higher-order surface currents and spreading energy throughout the ground surface. The CA (Fig. 20a) shows tighter clustering in the resonance band, indicating greater modal stability at higher frequencies. The EV (Fig. 20b) trends indicate clearly defined dominant modes, whereas the MS responses (Fig. 20c) show larger and more potent resonance peaks, indicating effective bandwidth enhancement. Thus, the defective ground surface enhances high-frequency modal coupling and promotes steady wideband radiation behaviour.

**Fig. 21:**
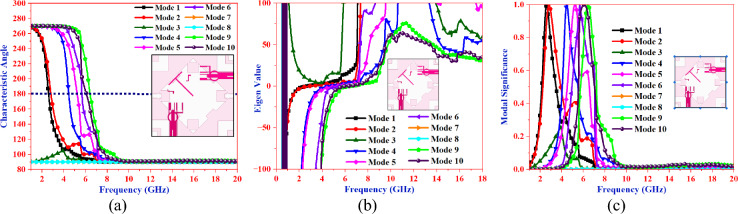
Modal analysis results for Stage 7 at 1.6 GHz (**a**) Characteristic Angle (**b**) Eigenvalue. (**c**) Modal Significance.

Figure 21 shows that the redesigned DGS pattern improves modal stability in Stage 7 (proposed structure) at 1.6 GHz. The CA (Fig. 21a) shows a more consistent transition to the 180° resonance zone, indicating stronger current channeling around the modified ground boundaries. The EV graphs (Fig. 21b) show that the dominant mode approaches resonance with reduced interference from surrounding modes, producing clearer radiation. Similarly, the MS curves (Fig. 21c) indicate more intense and discrete excitation of the fundamental mode, indicating improved low-frequency efficacy compared to previous stages.

**Fig. 22:**
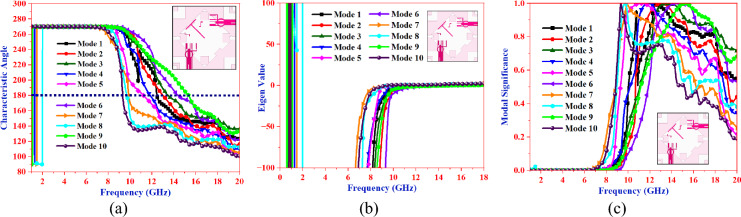
Modal analysis results for Stage 7 at 12.6 GHz (**a**) Characteristic Angle (**b**) Eigenvalue. (**c**) Modal Significance.

At 12.6 GHz (Fig. 22), the fully optimized defective ground structure enclosing the rotated square slot allows for a broader modal response. The CA (Fig. 22a) dispersion develops less abrupt, indicating smoother stimulation of several higher-order modes over the upper band. The EV plots (Fig. 22b) show closer mode grouping at the resonant area, indicating multi-modal emission behaviour with regulated interaction. The proposed configuration successfully improves high-frequency coupling and expands the radiation bandwidth, as further confirmed by MS (Fig. 22c), which shows that several modes exceed the 0.707 threshold.

In general, the three structural components; rotated square ground-plane etching, dual asymmetric slot-based feed structures, and the gradual introduction of defected-ground features (DGFs)—have the most influence on the CM throughout the design trajectory from Stage 1 to Stage 7. The 45° tilted square etch introduced in Stage 5 causes substantial redistribution of surface currents, allowing for strong disruption of fundamental modes and selective augmentation or suppression of orthogonal modal pairings. Peripheral DGFs are added in Stage 6, altering the boundary constraints along the ground surface. This enhances resonance alignment of higher-order modes in the uppermost band (≈12.6 GHz) and reduces their EVs. The most significant impact is seen in Stage 7, where the slanted ground etch, revised slots, and broader DGSs interact to deliver a wider range of strongly stimulated modes and enhanced modal significance profiles. Thus, the combination of ground-plane reconfiguration and slot perturbations—particularly in Stages 5–7 has the greatest influence on the modal behaviour.

### Parametric analysis

Tuning or optimization in antenna design is crucial because theoretical proportions rarely provide optimal efficiency across practical frequency ranges and applications. Small modifications to antenna components, such as feed gap, patch length, ground plane shape, and substrate architectural design, significantly affect resonance frequencies, impedance bandwidth, radiation efficiency, and isolation. Without tuning, the antenna could demonstrate mismatched impedance, undesirable resonances, or inadequate isolation in multi-element systems, resulting in poor performance in practical situations. Designers may tweak current distribution, dampen surface waves, and attain stable wideband performance utilizing systematic optimization approaches such as parametric sweeping, structural alterations, or the inclusion of parasitic and slot components. This technique assures that the antenna complies with theoretical standards, but also fulfils practical needs such as excellent isolation, low return loss, compact size, and sturdiness for contemporary uses such as MIMO, UWB, and 5G.

A systematic optimization technique was used to guarantee that the proposed antenna exhibits the required electrical and radiation parameters while remaining compact. Several design facets, including radiator geometry and arrangement, ground plane alterations, and parasitic element deployment, were iteratively refined. This parametric refinement not only allowed for exact control over impedance matching and bandwidth, but it additionally enhanced isolation among the radiating components resulting in stable performance across the targeted frequency range.

**Fig. 23:**
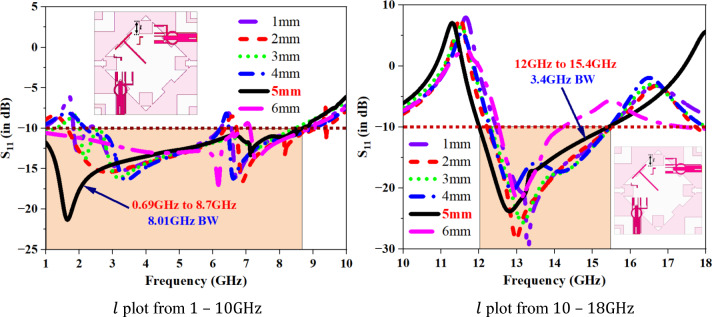
${\mathrm{S}}_{11}$ plot for change in the length of square stub (${\mathrm{l}}$) at the ground plane.

As shown in Fig. 23, tweaking ‘${\mathrm{l}}$’ significantly influences the ${\mathrm{S}}_{11}$ response, indicating its impact on current distribution and mutual coupling stabilization. Insufficient coupling at smaller strip lengths (1–3 mm) results in inadequate impedance matching and limited operational bandwidths. Excessive extension (≥ 6 mm) leads to over-coupling and low performance. The optimal value of l = 5 mm achieves a balanced EM interaction among the antennas and the parasitic patch, resulting in an ultra-wideband spectrum of 0.69—8.7 GHz (8.01 GHz bandwidth) in the lower band and an uninterrupted higher-order resonance with 3.4 GHz bandwidth (12—15.4 GHz) in the higher band.

**Fig. 24:**
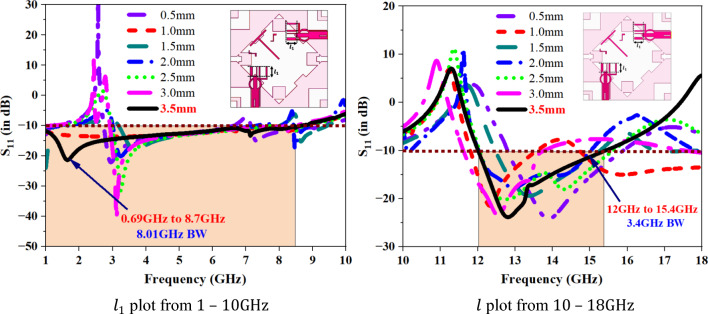
${\mathrm{S}}_{11}$ plot for change in the length of asymmetrical stub (${\mathrm{l}}_{1}$) on the radiationg patches.

In Fig. 24, it becomes apparent that shorter ‘l1’ lengths (0.5—1.5 mm) failed to achieve acceptable impedance matching over the entire band, resulting in sharp dips and limited bandwidth. As the frequency rises, the resonance turns more profound and wider, with the best instance happening at 3.5 mm (black curve). Similarly, in the second plot (12—15.4 GHz), parameter modification has a significant impact on the higher resonant phase. Smaller l1 values cause resonance shifts and poor coupling, leading to insufficient bandwidth. However, at 3.5 mm, the antenna has a well-defined resonance, deeper return loss, and a wider 3.4 GHz bandwidth (12—15.4 GHz).

**Fig. 25:**
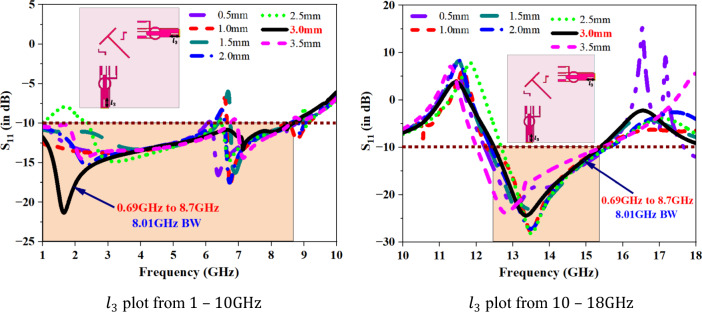
${\mathrm{S}}_{11}$ plot for change in the length of slot (${\mathrm{l}}_{3}$) on the radiationg patches.

**Fig. 26:**
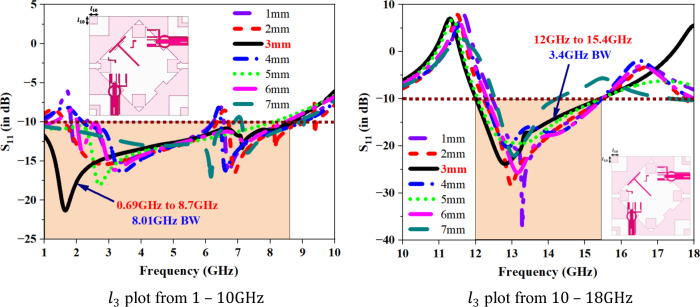
${\mathrm{S}}_{11}$ plot for change in the length of corner square stub (${\mathrm{l}}_{10}$) at the ground plane.

**Fig. 27:**
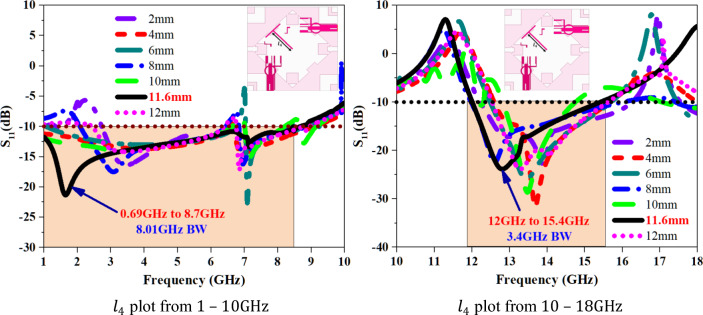
${\mathrm{S}}_{11}$ plot for change in the length of T-shape tail (${\mathrm{l}}_{4}$) on the radiationg patches.

The Fig. 25 and 26 show that changing ‘l3’ and ‘l10’ will marginally shifts the resonance frequencies and matching levels, emphasizing its purpose in fine-tuning the antenna behaviour. The modification of l4 (Fig. 27) causes minor alterations in the ${\mathrm{S}}_{11}$ plots and depth of the plot, but the overall wideband behavior remains intact. In the second plot, spanning 10 GHz to 18 GHz, the antenna exhibits another unique resonance region from roughly 12 GHz to 15.5 GHz. ${\mathrm{S}}_{11}$ values drop as low as −30 dB, confirming excellent impedance matching and efficient radiation at higher frequencies. The effect of adjusting the slot length between 2 and 12 mm is primarily to tune the resonance places and depths, allowing for design freedom while preserving dual-band functionality. The optimization parameters ${\mathrm{l}}$, ${\mathrm{l}}_{1}$, ${\mathrm{l}}_{3}$, ${\mathrm{l}}_{10}$, and ${\mathrm{l}}_{4}$ were chosen based on preliminary sensitivity assessment and physical rationale concerning their direct impact on resonances, ground-boundary constraints, and feed coupling.

## Results and discussions

This section provides a comprehensive analysis of the electromagnetic field dispersion surrounding the proposed antenna configuration, which provides insight into the radiation process and coupling behavior between the radiating components. Additionally, the design is thoroughly validated by comparing simulated and measured results for essential scattering characteristics (${\mathrm{S}}_{11}$ and ${\mathrm{S}}_{12}$), assuring impedance matching and inter-element isolation. Additionally, the MIMO diversity performance metrics, including ECC, DG, TARC, and MEG, are examined to validate the feasibility of the proposed antenna for UWB MIMO applications.

**Fig. 28:**
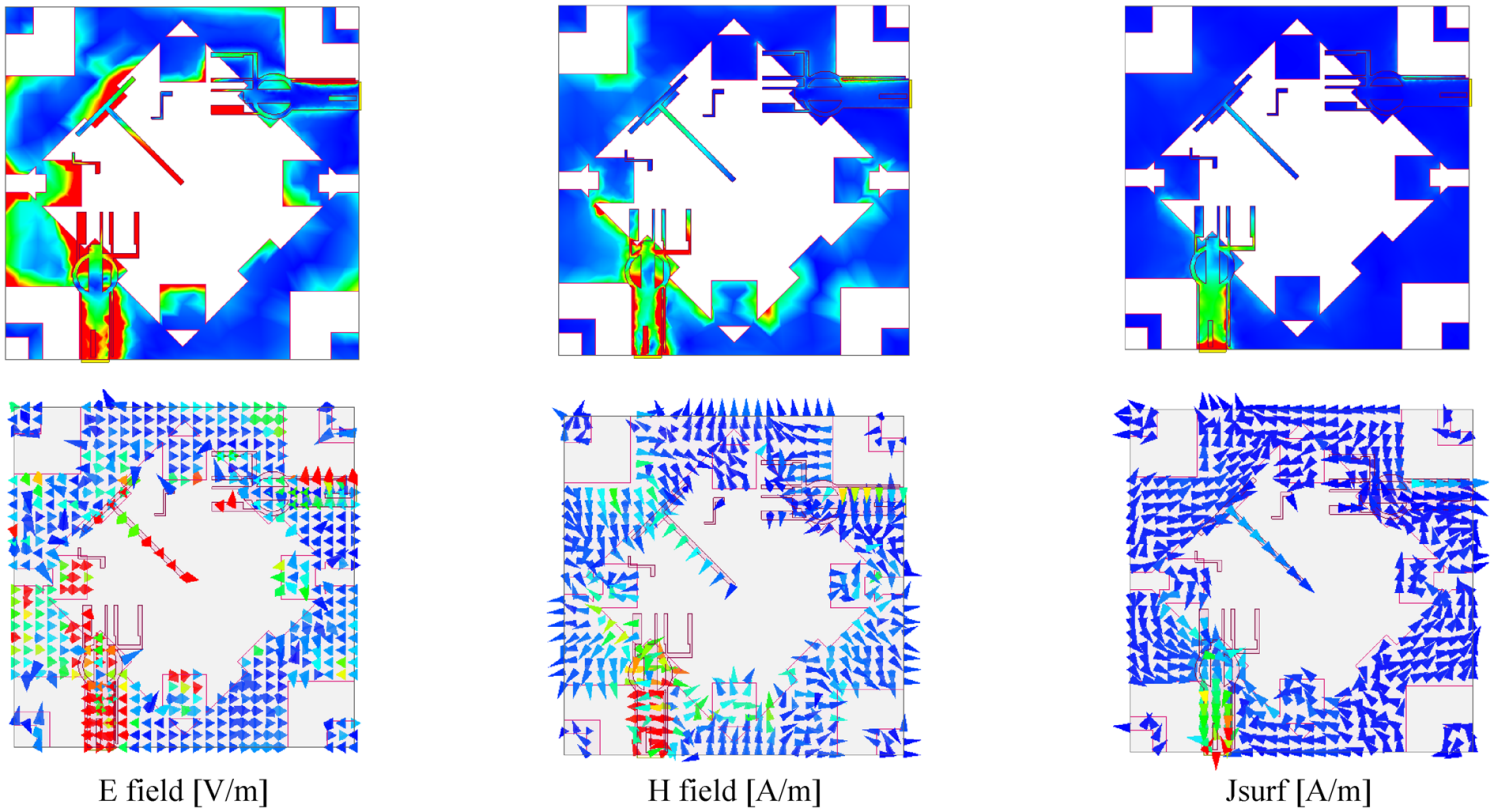
Field distribution around the two port MIMO antenna at 1.6GHz.

**Fig. 29:**
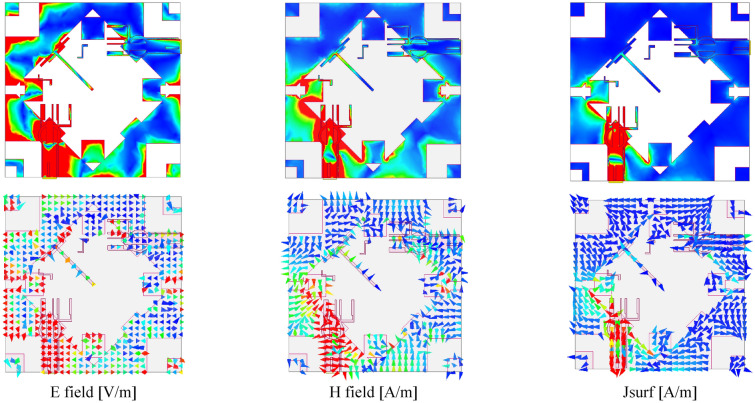
Field distribution around the two port MIMO antenna at 13GHz.

**Fig. 30:**
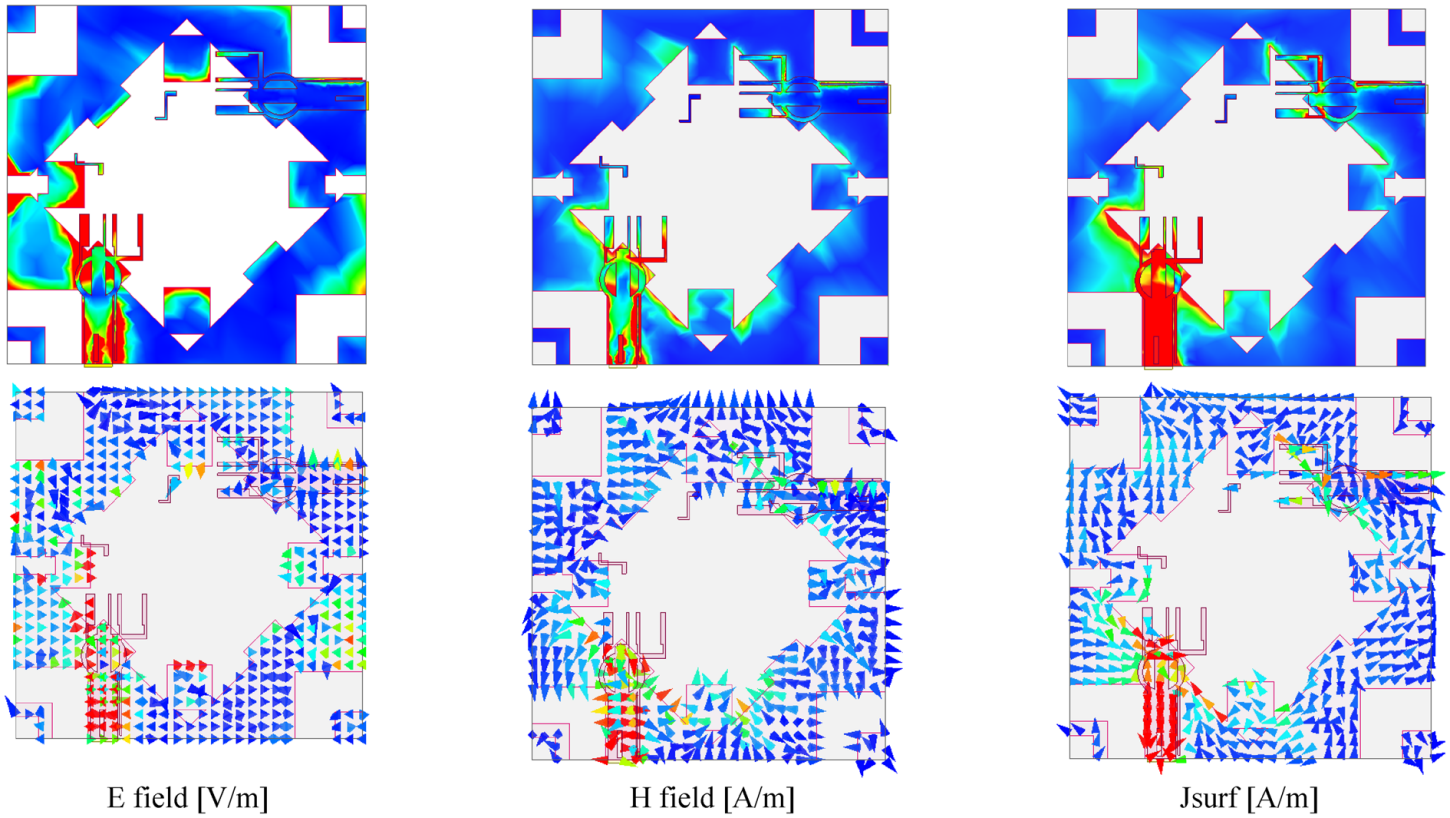
Field distribution around the two port MIMO antenna with out T-shape parasitic element at 1.6GHz.

**Fig. 31:**
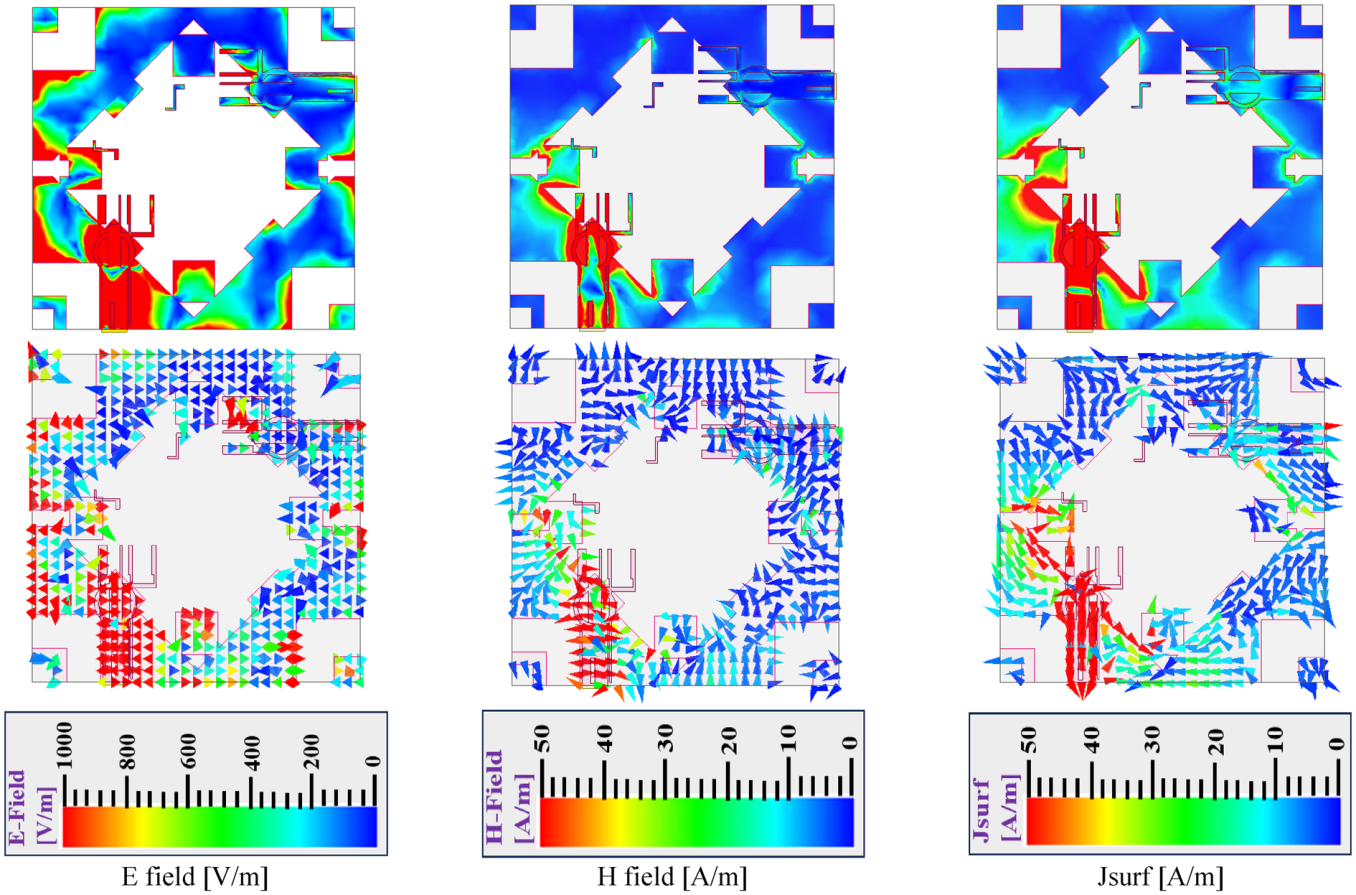
Field distribution around the two port MIMO antenna with out T-shape parasitic element at 13GHz.

Figures 28 and Fig. 29 represent the E-field, H-field, and surface current distributions on the proposed antenna at 1.6 and 13 GHz, respectively. Figures 30 and Fig. 31 demonstrate comparable field distributions of the structure without the T-shaped parasitic element, exhibiting it affects coupling suppression, current redistribution, and overall radiation performance.

As shown in Fig. 28, the significant electric field intensity along the T-shaped parasitic strip and radiating arm edges at 1.6 GHz, which is centred around the feed locations of the orthogonal radiators, confirms effective excitation and coupling suppression. While confinement close to the feeds implies resonance, the magnetic field fosters orthogonal loops around the radiators, allowing for polarization diversity. The defective ground slots redirect return currents to limit surface waves and increase isolation, whilst the T-shaped strip and radiator edges contain strong surface currents that disperse currents to reduce mutual coupling. The radiator-T-strip-ground configuration enables strong excitation, minimal coupling, and stable radiation performance with polarization diversity at this frequency. At 13 GHz (Fig. 29), however, the electric field distribution becomes significantly denser, with substantial localization at the radiator edges, parasitic strip, and defective ground, indicating the stimulation of higher-order modes. The magnetic field loops become more complex, indicating many resonant pathways, and the surface current density rises substantially, spreading across radiators, parasitic strip, and slots. The results obtained exhibit that, while the antenna maintains polarization diversity across frequencies, the transition from 1.6 GHz to 13 GHz shifts the operation from fundamental to higher-order resonances, with improved field confinement and stronger coupling suppression enabled by the combined radiator-parasitic-ground configuration.

Figures 30 and Fig. 31 demonstrate the surface current and field distributions of the proposed antenna at 1.6 GHz and 13 GHz without the T-shaped elements. At 1.6 GHz, the absence of the strip causes elevated electric field couplings between the orthogonal radiators, intersecting magnetic loops, and concentrated stream of current across both radiators, resulting in reduced isolation. At 13 GHz, the implications become more significant, as higher-order resonances generate strongly localized but unregulated electric fields, many overlapping magnetic loops, and prolonged current distribution across the radiators and ground plane, therefore increasing mutual interference.

These findings prove that the presence of T-shaped element is critical to lowering inter-element coupling, restricting currents, and retaining stable isolation throughout both primary and higher-level resonances.

**Fig. 32:**
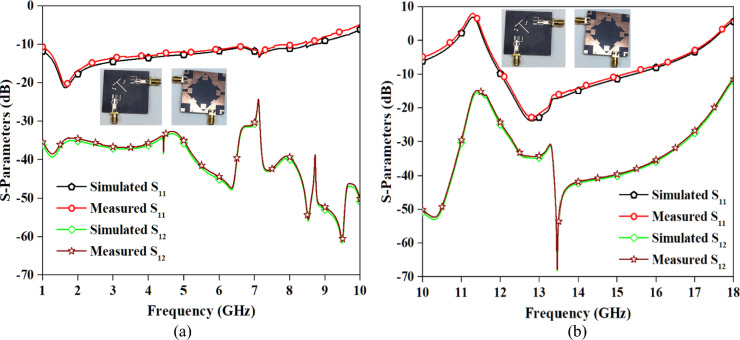
Simulated and Measured ${\mathrm{S}}_{11}$ plot ${\mathrm{S}}_{21}$ plot for the proposed antenna.

The computed and observed S-parameter findings for the proposed two-port antenna as illustrated in Fig. 32, shows dual-band wideband functioning with excellent port isolation (Fig. 32a and b). The observed ${\mathrm{S}}_{11}$ degradation beyond 6 GHz is mostly due to manufacture tolerances, where minor changes in slot and feed size create significant shifts at higher frequencies. Variations in substrate properties, such as ${\upvarepsilon }_{{\mathrm{r}}}$ tolerance and loss dispersion, can affect the practical electrical length. Furthermore, SMA connector and solder-joint parasitic cause mismatch, which becomes more noticeable in the 6–10 GHz range. These combined deviations explain the observed difference between simulation and measurement. The transmission coefficient (${\mathrm{S}}_{21}$) is below −20 dB and even approaching −40 dB at certain bands. This indicates high isolation amongst the two antennas, which is essential for MIMO operations.

### MIMO diversity parameters

In practice, using several antennas in a MIMO system improves signal quality and data rate by using multipath fading. However, antenna elements are connected to one another due to mutual coupling, correlation, and uneven power distribution. Therefore, examining reflection coefficient (${\mathrm{S}}_{11} < - 10{\mathrm{dB}}$) or gain alone does not guarantee MIMO efficiency. As a result, in MIMO configurations, diversity characteristics play a crucial role, with each addressing a distinct element of how numerous antennas interact.

#### Envelope correlation coefficient (ECC)

The ECC sketch assesses the diversity functionality of the proposed design. ECC is an important statistic in MIMO antennas because it quantifies the relationship between the radiation patterns of the antennas; lower ECC quantities show better isolation and more diversity gain. For MIMO system, ECC ought to be below 0.5, with values close to 0 being preferred.

Estimation of ECC using S-parameters:

For a two-port antenna, an approximate ECC can be computed using S-parameters using the following formula:

14 $${\uprho } = \frac{{\left| {{\mathrm{S}}_{11} {\mathrm{S}}_{12}^{*} + {\mathrm{S}}_{21} {\mathrm{S}}_{22}^{*} } \right|^{2} }}{{\left( {1 - \left| {{\mathrm{S}}_{11} } \right|^{2} - \left| {{\mathrm{S}}_{21} } \right|^{2} } \right)\left( {1 - \left| {{\mathrm{S}}_{22} } \right|^{2} - \left| {{\mathrm{S}}_{12} } \right|^{2} } \right)}}{ }$$

Calculation of ECC using far-field patterns:

15 $$\rho = \frac{{\left| {{ iint }F_{1} \left( {\hat{r}} \right).F_{2}^{*} \left( {\hat{r}} \right)d\Omega } \right|^{2} }}{{\left( {{ iint }\left| {F_{1} \left( {\hat{r}} \right)} \right|^{2} d\Omega } \right)\left( {{ iint }\left| {F_{2} \left( {\hat{r}} \right)} \right|^{2} d\Omega } \right)}}$$

where, ${\mathrm{F}}_{{\mathrm{i}}}$ denotes the far-field complex vectors.

**Fig. 33:**
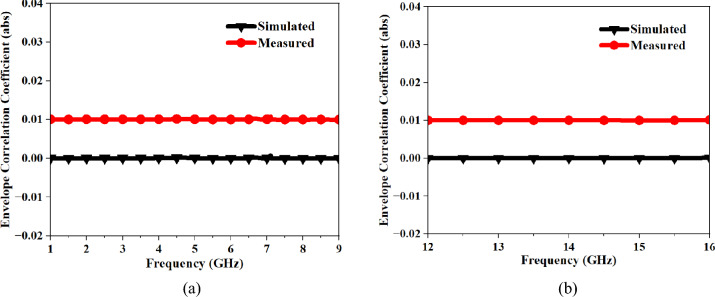
Simulated and Measured ECC plot for the proposed antenna (**a**) ECC response from 1–9GHz (**b**) ECC response from 12–16GHz.

The Fig. 33 shows that both measured and simulated ECC values are consistently low across the full working frequency span of 1–18 GHz (Fig. 33a and b), with simulation results very close to and measured readings around 0.01. These values are much lower than the 0.5 threshold, indicating good diversity performance and negligible correlation among antenna elements.

#### Diversity gain (DG)

The DG is a significant metric in MIMO antenna since it measures how well the antenna enhances signal stability and reduces multipath fading. An efficient MIMO communications system should have DG close to 10 dB to ensure excellent diversity performance. The value of DG is computed using the following formula:

16 $${\mathrm{DG}} \approx 10\sqrt {1 - {\uprho }^{2} } { }\left( {{\mathrm{dB}}} \right)$$

**Fig. 34:**
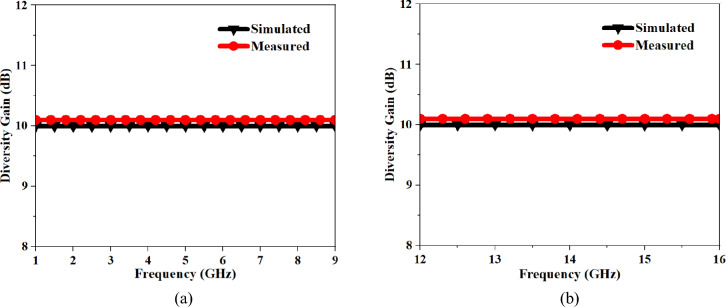
Simulated and Measured ${\mathrm{DG}}$ plot for the proposed antenna (**a**) DG response from 1–9GHz. (**b**) DG response from 12–16GHz.

Figure 34 illustrates the computed and evaluated results of the DG. In this plot, both the simulated and measured DG values remain consistently close to 10 dB across the entire band (Fig. 34a and b), with the simulated values slightly below and the measured values slightly above 10 dB. This negligible deviation confirms excellent agreement between simulation and measurement. The recorded DG implies that the proposed structure delivers exceptional diversity performance while minimising degradation, facilitating stable operation in multipath circumstances.

#### Channel capacity loss (CCL)

The parameter CCL computes the loss or deviation in system capacity due to inefficiency or correlation. For a $2 \times 2$ correlation matrix, the CCL is theoretically calculated using the following expression:

17 $${\mathrm{CCL}} = - \log_{2} \left( {\det \left( {\mathrm{R}} \right)} \right){\text{ with R}} = \left[ {\begin{array}{*{20}c} 1 & {\uprho } \\ {{\uprho }^{*} } & 1 \\ \end{array} } \right]$$

where, $\det \left( {\mathrm{R}} \right) = 1 - \left| {\uprho } \right|^{2}$, hence the Eq. (4) becomes

18 $${\mathrm{CCL}} = - \log_{2} \left( {1 - \left| {\uprho } \right|^{2} } \right){ }\left( {{\mathrm{bits}}/{\mathrm{s}}/{\mathrm{Hz}}} \right)$$

**Fig. 35:**
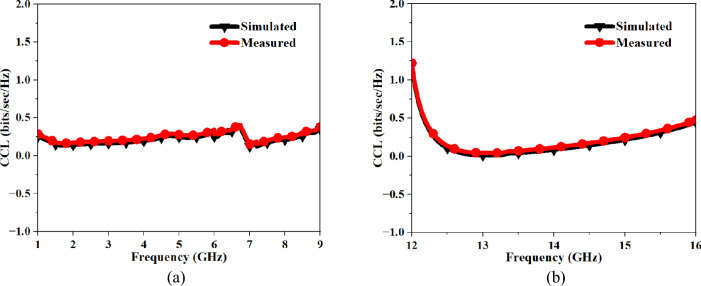
Simulated and Measured ${\mathrm{CCL}}$ plot for the proposed antenna (**a**) CCL response from 1–9GHz. (**b**) CCL response from 12–16GHz.

Figure 35 depicts the computed and actual CCL of the proposed MIMO antenna. Over the lower frequency spectrum (less than 10 GHz, as depicted in Fig. 35a), the CCL is considerably below the crucial level of 0.4 bits/sec/Hz, indicating substantial port isolation and negligible correlations among radiating parts. At larger frequencies (12–16 GHz, as depicted in Fig. 35b), computed and actual responses follow similar trends. The high degree of agreement between the results confirms that the antenna configuration is effective, and the reduced CCL across most of the working spectrum increases channel capacity while guaranteeing stable MIMO operation.

#### Mean effective gain (MEG)

The MEG is an important diversity performance statistic in MIMO antennas, guaranteeing that each antenna element receives approximately equal average power under multipath situations. For the perfect MIMO system, the disparity in MEG values across the two ports should be less than 3 dB, and the MEG/Power Ratio should be close to unity (0 dB). In a uniform environment, MEG can be computed using the following expression:

19 $${\mathrm{MEG}}_{{\mathrm{i}}} = \frac{1}{2}\left( {1 - \left| {{\mathrm{S}}_{{{\mathrm{ii}}}} } \right|^{2} - \mathop \sum \limits_{{{\mathrm{j}} \ne {\mathrm{i}}}} \left| {{\mathrm{S}}_{{{\mathrm{ij}}}} } \right|^{2} } \right)$$

**Fig. 36:**
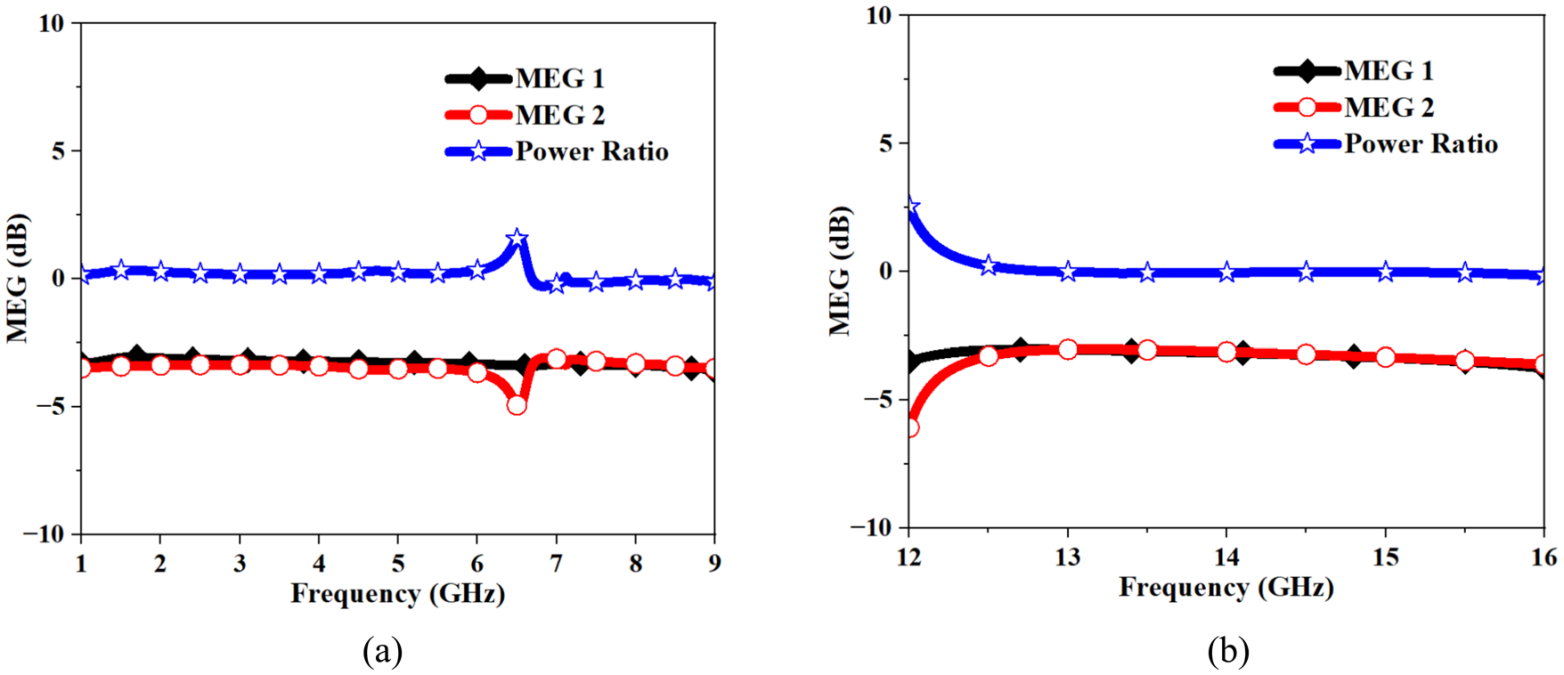
Simulated ${\mathrm{MEG}}$ and Power Ratio plot for the proposed antenna (**a**) MEG response from 1–9GHz. (**b**) MEG response from 12–16GHz.

As depicted in Fig. 36, the recorded value show that MEG 1 and MEG 2 are around −5 dB in both the lower (1–9 GHz, as illustrated in Fig. 36a) and higher frequency range (12–16 GHz, as illustrated in Fig. 36b). The approximately synchronous behaviour of MEG 1 and MEG 2 suggests consistent power receipt across both ports, resulting in good diversity efficacy. The MEG variation among the two ports remains within the permissible 3 dB limit, which meets the MIMO design standards.

The power ratio line remains very near to 0 dB across the operational regions, indicating equal power dispersion across the two antennas. The small fluctuations at particular frequencies (at 6.5 GHz in the lower band) are due to the particular band-notched behaviour, although the variations are insignificant and do not impair system efficiency in general. As a result, the MEG and power ratio study confirm that the proposed structure offers balanced performance and outstanding diversity gain across the two ports. Equation (19) assumes an isotropic, and unpolarized multipath setup, where the MEG of each port equals half of its embedded radiation efficiency. In realistic propagation conditions which are not isotropic or totally unpolarized, the MEG must be assessed using the generalized form provided in Appendix A.

#### Total active reflection coefficient (TARC)

The TARC defines the overall system reflection when all ports are simultaneously excited instead of 1 port at a time. It captures mutual coupling effect in multi-port excitation. For a two-port antenna with equal excitation, the TARC can be computed using the following formula:

20 $${\mathrm{TARC}} = \sqrt {\frac{{\left| {{\mathrm{S}}_{11} + {\mathrm{S}}_{12} } \right|^{2} + \left| {{\mathrm{S}}_{21} + {\mathrm{S}}_{22} } \right|^{2} }}{2}}$$

**Fig. 37:**
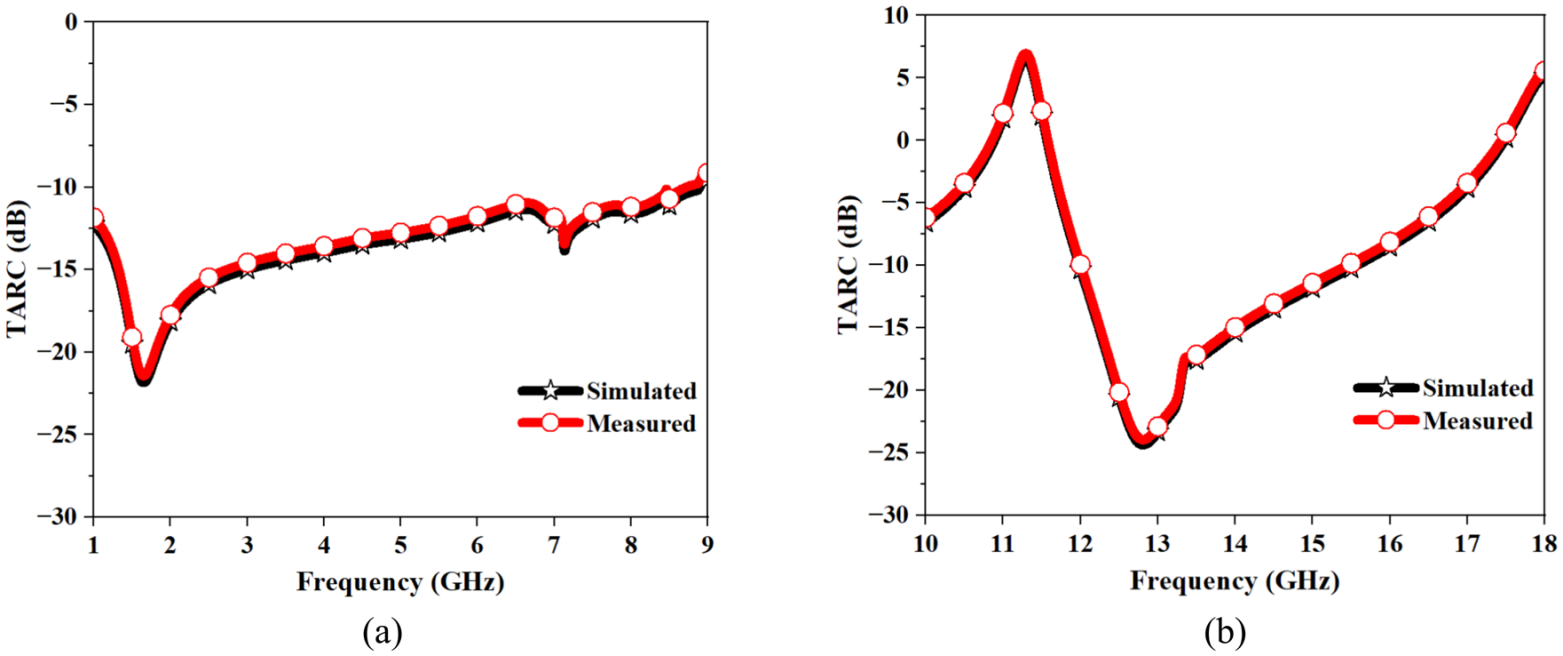
Simulated and Measured TARC plot for the proposed antenna (**a**) TARC response from 1–9GHz. (**b**) TARC response from 12–16GHz.

Figure 37 shows the computed and measured TARC of the proposed antenna over the frequency span between 1–18 GHz. TARC is an important metric in MIMO systems since it accounts for the cumulative reflection performance of all ports when multiple excitations with varied phase limitations are applied concurrently. Compared to traditional ${\mathrm{S}}_{11}$ or ${\mathrm{S}}_{22}$ analysis, TARC delivers a more accurate estimate of the active impedance matching of the radiators in MIMO operation.

As illustrated, the recorded TARC is consistently less than −10 dB across the bulk of the operational bandwidth, indicating that the antenna has excellent impedance matching under active port excitations. As illustrated in Fig. 37a (1–9 GHz), TARC values fall below −20 dB near 2 GHz and stay far below the −10dB barrier throughout the lower UWB range, ensuring efficient radiation and minimal mismatch losses. Similarly, Fig. 37b (10–18 GHz) demonstrates that the TARC maintains values as low as −25 dB about 12 GHz, with just a small area of enhanced reflection induced by the anticipated band-notch behaviour. The strong agreement between anticipated and measured curves confirms the accuracy of the design and fabrication processes, with only minor differences due to connector losses and measurement setup tolerances.

**Fig. 38:**
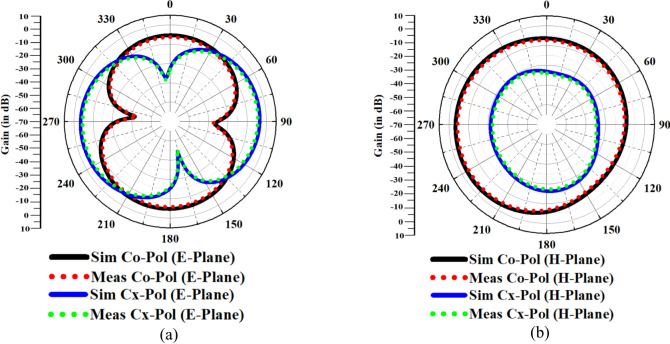
Simulated and Measured Co and X polarization plot (**a**) for E plane at 1.6GHz (**b**) H – plane at 1.6GHz.

**Fig. 39:**
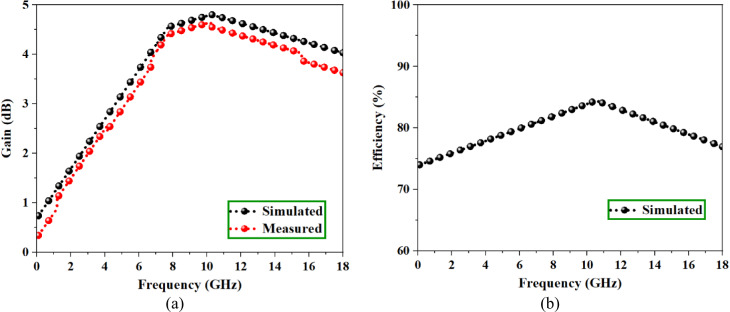
Recorded Gain and Efficiency Characteristics (**a**) Simulated and Measured Gain plot (**b**) Simulated Radiation Efficiency.

The simulated and measured E – (Co and Cross) and H – plane (Co and Cross) radiation patterns are shown in Fig. 38. The Co – polarization plot shows a broader radiation pattern along with stable gain, and the corresponding X – polarization recorded value is substantially smaller in magnitude than the Co – Pol response, demonstrating excellent polarization purity. The occurrence of deep nulls at ± 90° are further confirmed better radiation directivity and low polarization leakage. In the H – plane plot, the X – Pol levels remained much lower than the Co – Pol levels along the angular spectrum, exhibiting the radiator capacity to preserve polarization integrity over the entire H – plane, Fig. 39.

The proposed antenna exhibits a gradual rise in gain from roughly 0.5 dB at 1 GHz to an optimum value of around 4.9 dB around 10–12 GHz, preceding a slight fall approaching 18 GHz. The observed decline between 8 and 10 GHz indicates a decline in impedance matching, as the reflection coefficient reaches the −10 dB barrier, reducing power provided to the radiator. Since the gain achieved is determined by

21 $${\mathrm{G}}_{{{\mathrm{real}}}} \left( {\mathrm{f}} \right) = {\upeta }_{{{\mathrm{rad}}}} \left( {\mathrm{f}} \right){\mathrm{D}}\left( {\mathrm{f}} \right)$$

and the radiation efficiency follows

22 $${\upeta }_{{{\mathrm{rad}}}} \left( {\mathrm{f}} \right) = \left[ {1 - \left| {{\Gamma }\left( {\mathrm{f}} \right)} \right|^{2} } \right]{\upeta }_{{{\mathrm{int}}}} \left( {\mathrm{f}} \right)$$

where, ${\Gamma }\left( {\mathrm{f}} \right)$ is the frequency-dependent reflection coefficient, and ${\upeta }_{{{\mathrm{int}}}}$ is the intrinsic efficiency of the radiator.

This correlation is visible in the measured drop, in which mismatch losses and decreased efficiency converge. Furthermore, near the upper edge of the main UWB resonance spectrum (8–10 GHz), the antenna shifts to higher-order current modes, becoming more susceptible to structural asymmetry caused by the offset feeding and ground-surface slots. These modal shifts disperse surface currents, which reduces efficiency and contributes to transient gain degradation. Beyond this range, the gain constantly exceeds 4 dB up to 18 GHz, implying high radiation efficiency and reliable broadband efficacy. The strong match between predicted and measured gain plots verifies the feasibility of the antenna design for wideband and multiband communication applications.

The proposed layout is benchmarked against existing literature, and the performance characteristics are systematically summarised in Table 2.

**Table 2:** A Comparison study of the proposed structure with the available literature.

Ref	Year	No. of Ports	Antenna Size (${\boldsymbol{\lambda}}$)	IAS	IB (in GHz)	IT |${\boldsymbol{S}}_{21}$|	I	PG	ECC, DG (dB)	TARC (dB)	MEG (dB)	CCL (bits/s/Hz)
^10^	2020	2	$0.74 \times 0.37\lambda^{2}$	8	3.1–10.6	MTM	17	4.9	0.02, –	–	–	0.5
^39^	2020	2	$0.15 \times 0.24\lambda^{2}$	2	2.8–14.45	Stubs and PTL	22	6.86	0.08, 9.9	–10	–	–
^40^	2020	3	$0.48 \times 0.96\lambda^{2}$	–	3.3–3.8	Quasi- Yagi	20	7.38	0.001, 9.99	–	–	–
^41^	2020	4	$0.29 \times 0.29\lambda^{2}$	5	3.4–3.8	Mirroring	18	5	0.1, –	–	–	–
^42^	2021	2	$0.57 \times 0.39\lambda^{2}$	5	3–7.70	Comb shape	20	3	0.04 9.98	–10	–3	0.3
^43^	2021	2	$0.96 \times 0.48\lambda^{2}$	N.R	2.0–2.7 3–4.27	Self-isolation	12	4.2	0.0056,10	N.R	N.R	10
^44^	2021	4	$0.43 \times 0.43\lambda^{2}$	4.2	3.2–3.68 4.8–5.0	Parasitic Ground stub	16	4.4	0.025, –	N.R	N.R	N.R
^45^	2022	2	$0.19 \times 0.19\lambda^{2}$	N.R	18.9–34.8	Self-isolation	35	N.R	0.005, 9.99	–10	N.R	0.2
^46^	2022	2	$0.48 \times 0.48\lambda^{2}$	N.R	3.38–5.1	Polarization	22.5	7	0.03, –	N.R	N.R	N.R
^47^	2023	2	$0.23{ } \times 0.40\lambda^{2}$	N.R	2.95–6.3	Ground stub	15	3	0.012, 10	–10	–3	0.05
^48^	2023	2	$0.24{ } \times 0.42\lambda^{2}$	N.R	3.4–3.6	U, T –stubs	18	2.72	N.R	N.R	N.R	N.R
^12^	2025	2	$0.56{ } \times 1.35\lambda^{2}$	N.R	28–38	MTM	25	5.2	0.0001, 10	–22	N.R	0.05
^49^	2024	2	$0.45{ } \times 0.27\lambda^{2}$	8.5	2–18	Parasitic	25	6.5	0.001, 10	N.R	–9	0.30
^11^	2024	2	$0.23{ } \times 0.36\lambda^{2}$	1.5	3.25–3.85	Stubs and PTL	19	3.259	0.011, 10	–10	0.481	0.30
^27^	2024	4	$0.54{ } \times 0.54\lambda^{2}$	8	3–4.12	Self-isolation	21	4	0.005, 9.9	–5	–3	0.2
^50^	2025	2	$0.47{ } \times 1\lambda^{2}$	16	3.7–7.60	Parasitic	21	4.6	0.05, 10	N.R	−3	0.3
**Proposed**		**2**	$0.2\user2{ } \times 0.2{\boldsymbol{\lambda}}^{2}$	**–**	**0.7–8.6** **12–15.4**	**Inverted T stub**	**30**	**4.8**	**0.01, 10**	**–25**	**–3**	**0.3**

IAS: Inter Antenna Spacing IB: Impedance Bandwidth IT: Isolation Technique I: Isolation PG: Peak Gain ECC: Envelope Correlation Coefficient DG: Diversity Gain TARC: Total Active Reflection Coefficient MEG: Mean Effective Gain CCL: Channel Capacity Loss PTL: protruded transmission lines, N.R: Not Reported, MTM: Metamaterial.

## Conclusion

This study presents a miniaturized two-port UWB MIMO antenna integrating a band-notch mechanism within an extremely compact footprint of $38.5 \times 38.5 \times 0.5{\mathrm{mm}}^{3}$ ($0.2 \times 0.2 \times 0.002{\uplambda }^{3}$). The orthogonal radiator configuration, complemented by a 45° tilted T-shaped parasitic strip, enables polarization decorrelation and maintains port isolation better than −30 dB throughout the operational bands. The offset-fed technique and asymmetrical ground-plane slots jointly realize an ultra-wide impedance bandwidth, while simultaneously providing controlled band suppression.

The antenna supports dual resonances at 1.6 GHz and 12.6 GHz, delivering fractional bandwidths of 170.1% (0.7–8.6 GHz) and 24.82% (12.0–15.4 GHz), respectively, thereby covering GPS, ADS-B, UWB, X-band radar, and Ku-band satellite applications. Comprehensive performance analysis demonstrates a peak gain of 4.9 dB, ECC < 0.01, favorable MEG balance, and TARC values below –10 dB, confirming the antenna’s suitability for high-capacity, interference-resilient MIMO systems.

Overall, the proposed architecture offers a novel combination of compactness, wideband capability, strong isolation, and low correlation, positioning it as a viable candidate for advanced wireless, radar, and satellite communication platforms.

## Data Availability

The datasets used and/or analysed during the current study available from the corresponding author on reasonable request.

## Appendix

### Appendix A—generalized formulation for non-uniform multipath settings

In practical wireless channels, the inward field dispersion is often directional and may be partially polarized. In general, the MEG of port 'i' is

23 $$MEG_{i} = \smallint_{4\pi } P \left( {\theta , \phi } \right) M_{pol} \left( {\theta , \phi } \right) G_{i} \left( {\theta , \phi } \right) d\Omega$$

where ${\text{P }}\left( {{\uptheta },{ }\phi } \right)$ is the normalized PAS of the incident field (IF), $M_{pol} \left( {\theta , \phi } \right) \varepsilon \left[ {0,1} \right]$ is the polarization matching factor in between IF and the antenna polarization, and ${\mathrm{G}}_{{\mathrm{i}}} \left( {{\uptheta },{ }\phi } \right)$ is the embedded gain pattern of ‘i’ port when other ports are terminated in match loads.

For an isotropic and unpolarized environment, $${\text{P }}\left( {{\uptheta },{ }\phi } \right) = { }{\raise0.7ex\hbox{$1$} \!\mathord{\left/ {\vphantom {1 {4{\uppi }}}}\right.\kern-0pt} \!\lower0.7ex\hbox{${4{\uppi }}$}}$$ and $${\mathrm{M}}_{{{\mathrm{pol}}}} \left( {{\uptheta },{ }\phi } \right) = { }{\raise0.7ex\hbox{$1$} \!\mathord{\left/ {\vphantom {1 2}}\right.\kern-0pt} \!\lower0.7ex\hbox{$2$}}$$.

Substituting these in Eq. (23) yields

24 $$MEG_{i} = \frac{1}{2}\frac{1}{4\pi }\smallint_{4\pi } G_{i} \left( {\theta , \phi } \right) d\Omega = \frac{1}{2} \eta_{emb,i}$$

where, ${\upeta }_{{{\mathrm{emb}},{\mathrm{i}}}} = 1 - \left| {{\mathrm{S}}_{{{\mathrm{ii}}}} } \right|^{2} - \mathop \sum \limits_{{{\mathrm{j}} \ne {\mathrm{i}}}} \left| {{\mathrm{S}}_{{{\mathrm{ij}}}} } \right|^{2}$ is the embedded radiation efficiency, hence retreiving Eq. (6). To capture the realistic MEG in directed or polarized multipath, utilize Eq. (23), weighting the antenna gain by the observed or assumed PAS.

## Abbreviations

MIMO: Multiple input multiple output
UWB: Ultra-wideband
ECC: Envelope correlation coefficient
MEG: Mean effective gain
TARC: Total active reflection coefficient
CCL: Channel capacity loss
DG: Diversity gain
GPS: Global positioning system
ADS-B: Automatic dependent surveillance–broadcast
FCC: Federal communications commission
SNR: Signal-to-noise ratio
5G NR: 5G new radio
EBG: Electromagnetic bandgap
ML: Meander-line
MTM: Metamaterial
FBW: Fractional bandwidth
RC: Reflection coefficient
RS-DGS: Rotated square defected ground structure
EMI: Electromagnetic interference
NL: Neutralization lines
IoT: Internet of things
PAS: Power angular spectrum
MoM: Method of moments
